# RNA-Sequencing data supports the existence of novel VEGFA splicing events but not of VEGFA_xxx_b isoforms

**DOI:** 10.1038/s41598-017-00100-3

**Published:** 2017-03-03

**Authors:** Stephen Bridgett, Margaret Dellett, David A. Simpson

**Affiliations:** Centre for Experimental Medicine, Queen’s University Belfast, Belfast, Northern Ireland UK

## Abstract

Vascular endothelial growth factor (VEGFA), a pivotal regulator of angiogenesis and valuable therapeutic target, is characterised by alternative splicing which generates three principal isoforms, VEGFA_121_, VEGFA_165_ and VEGFA_189_. A second set of anti-angiogenic isoforms termed VEGFA_xxx_b that utilise an alternative splice site in the final exon have been widely reported, with mRNA detection based principally upon RT-PCR assays. We sought confirmation of the existence of the VEGFA_xxx_b isoforms within the abundant RNA sequencing data available publicly. Whilst sequences derived specifically from each of the canonical VEGFA isoforms were present in many tissues, there were no sequences derived from VEGFA_xxx_b isoforms. Sequencing of approximately 50,000 RT-PCR products spanning the exon 7–8 junction in 10 tissues did not identify any VEGFA_xxx_b transcripts. The absence or extremely low expression of these transcripts *in vivo* indicates that VEGFA_xxx_b isoforms are unlikely to play a role in normal physiology. Our analyses also revealed multiple novel splicing events supported by more reads than previously reported for VEGFA_145_ and VEGFA_148_ isoforms, including three from novel first exons consistent with existing transcription start site data. These novel VEGFA isoforms may play significant roles in specific cell types.

## Introduction

The pivotal role of VEGFA in angiogenesis has been recognised for many years^1^ and it is one of the most extensively studied growth factors. Inappropriate angiogenesis is a key factor in a range of conditions including cancer and proliferative eye diseases; blockade of VEGFA signalling to inhibit angiogenesis and reduce vascular leakage forms the basis of multiple effective clinical treatments^2^. Anti-VEGFA therapies can maintain and even improve visual acuity in neovascular age-related macular degeneration^3^.

**Figure 1:**
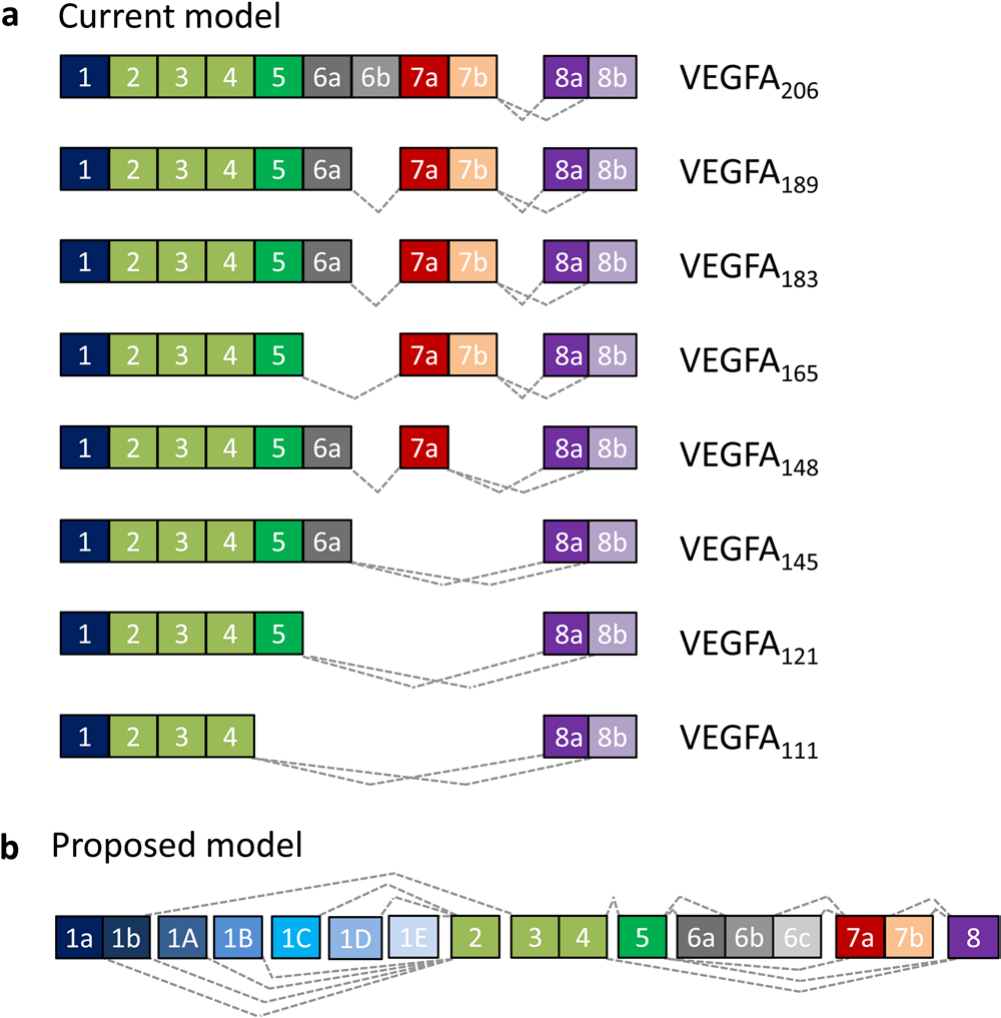
Overview of VEGFA splicing. In the current model exons 1-4 are present in all VEGFA mRNA transcripts. Inclusion or exclusion of different combinations of exons 5 to 7, or variants thereof, creates transcripts encoding VEGFA peptides of differing lengths (indicated by subscript numbers) and biological properties. It has been suggested that use of an alternative 3′ splice site for exon 8 can result in a shorter terminal exon (8b) encoding a peptide of the same length but with a different C-terminus which bestows anti-angiogenic properties. Numerous VEGFA_xxx_b variants have been reported, including VEGFA_111b_^55^; VEGFA_165b_^8^; VEGFA_121b_, VEGFA_183b_, VEGFA_145b_^34^; VEGFA_189b_^56^ and VEGFA_206b_^57^. Based upon the analysis of RNA-Seq data presented in this study, we propose an even more complex model for VEGFA transcription which includes alternative first exons and additional splicing events (a novel exon 6 splice site creates exon 6b and the existing exon 6b is renamed 6c). There is a single exon 8 with no splicing to the 8b site.

VEGFA provides a paradigm of how alternative splicing can give rise to a set of related proteins with shared domains and variable regions which confer differing biological properties^4–6^. The VEGFA splice isoforms (Fig. 1a) are named according to the number of amino acids they contain (e.g. VEGFA_121_ or VEGFA_165_). They differ in their bioavailability, the shorter isoforms being freely diffusible while the longer isoforms are highly basic and bound by the extracellular matrix^7^.

The report that splicing could occur to a downstream site within exon 8 (creating exon 8b) and generate an additional family of anti-angiogenic isoforms dubbed VEGFA_xxx_b^8^ added an intriguing further level of complexity to the VEGFA splicing story. Many publications have since described the presence of VEGFA_xxx_b transcripts *in vivo* and defined the anti-angiogenic properties of the proteins they encode^9–11^. Indeed it has been suggested that these isoforms are implicated in human diseases such as systemic sclerosis^10^, peripheral artery disease^9^ and maternal gestational diabetes^12^. These findings raise the alarming possibility that many studies of VEGFA need to be re-evaluated due to potential overlap between detection of VEGFA_xxx_ or VEGFA_xxx_b isoforms^13^, with potential implications for VEGFA-based therapies^14^.

All PCR-based assays are prone to artefacts due to the risk of amplification following hybridisation of primers to regions with incomplete complementarity and the validity of the RT-PCR assays which provide most of the evidence for the expression of VEGFA_xxx_b mRNA *in vivo* has been disputed^15^. In response it was suggested that specific experimental conditions and use of appropriate controls are critical for detection of VEGFA_xxx_b isoforms^16^. Although the VEGFA_xxx_b proteins have been overexpressed and demonstrated to be functional, for example reducing KDR (also known as VEGFR-2) phosphorylation^10^, this does not bear upon whether they are expressed *in vivo*. Antibodies raised against the VEGFA_xxx_b c-terminus have detected signals in tissues and blood^9, 10, 12, 17–19^ but this could be due to translational read-through of exon 8a-containing transcripts^20, 21^ or cross-reactivity.

If VEGFA_xxx_b isoforms exist *in vivo* then modulation of their expression provides a very attractive therapeutic approach. Indeed it has been suggested that the balance between pro- and –anti-angiogenic VEGFA_xxx_b isoforms synthesised by cancer cells could be targeted as a therapy^22^. VEGFA_xxx_b proteins have been demonstrated to have anti-angiogenic properties, but if they are not endogenous there is no rationale to limit investigations to these isoforms and other exogenous VEGFA variants may prove to be even more beneficial. It is therefore important to clarify whether VEGFA_xxx_b isoforms exist *in vivo*. High throughput next generation sequencing (NGS) has enabled the transcriptome to be investigated with unprecedented depth (eg ENCODE (Encyclopedia of DNA Elements) Consortium^23^ and GTEx^24, 25^) and we reasoned that if VEGFA_xxx_b isoforms do exist *in vivo* they should be represented in RNA sequencing data. Examination of RNA-Seq reads from a wide range of tissues and cancer samples revealed sequences derived from the canonical exon 8a-containing VEGFA isoforms in all datasets, but no sequences indicative of any VEGFA_xxx_b isoform. The absence of sequences spanning the proposed VEGFA exon 8b junction suggests that VEGFA_xxx_b transcripts do not exist or are expressed at extremely lowly levels *in vivo* and therefore that these isoforms are unlikely to play a significant role in normal physiology.

## Results

Reports of VEGFA_xxx_b transcripts have relied predominantly upon RT-PCR assays^8, 26^, which although very sensitive can be difficult to interpret due to the potential for mis-priming^15^. Seeking independent verification, we reasoned that if the putative VEGFA_xxx_b transcripts exist, they would be represented within the wealth of RNA sequencing data now publicly available^27^.

### Analysis of Expressed Sequence Tags (ESTs)

**Figure 2:**
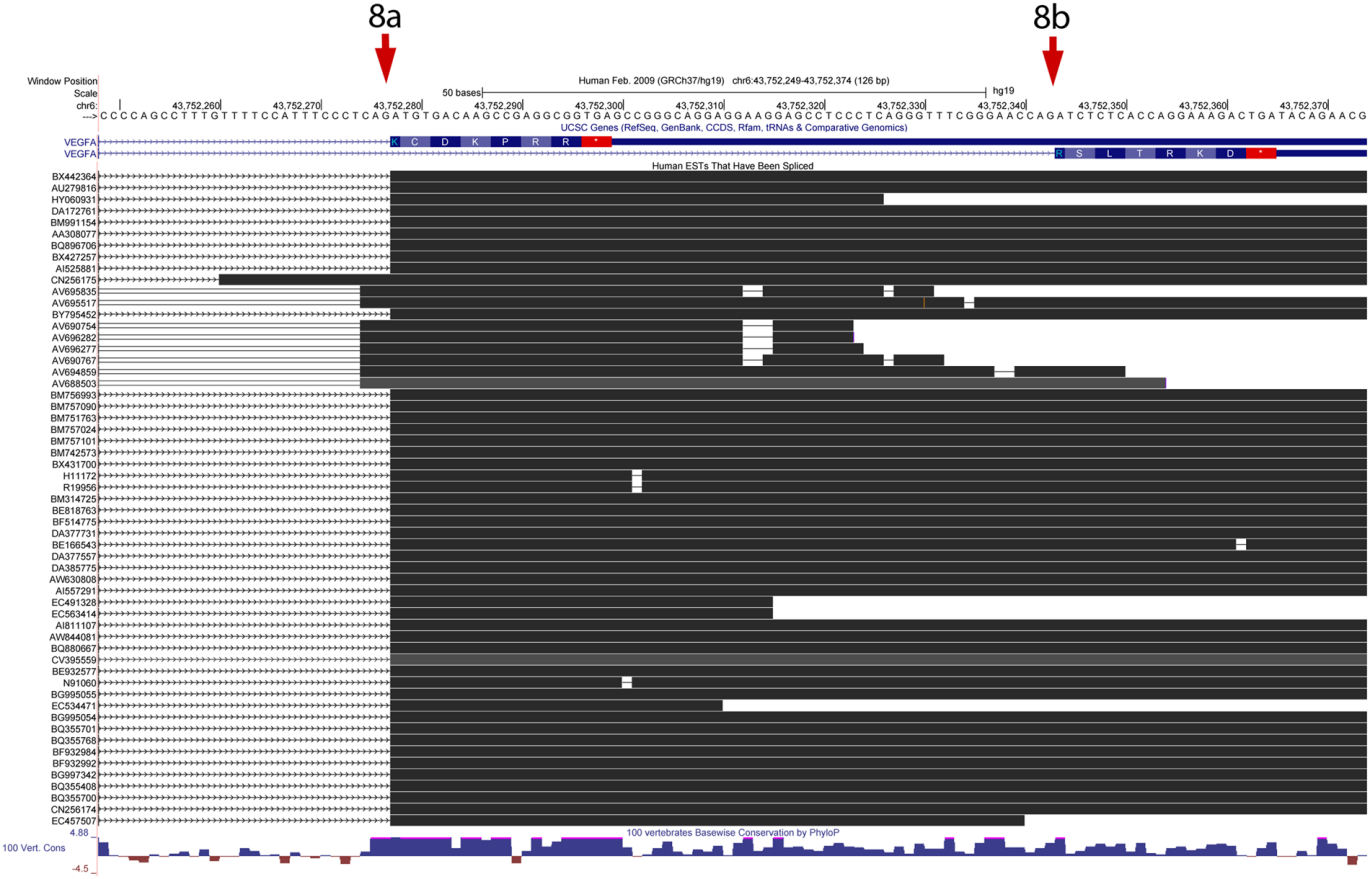
ESTs spanning the exon 8 splice junction viewed in the UCSC genome browser. All available spliced ESTs indicate the exclusive use of exon 8a, with none supporting the existence of exon 8b.

For many years ‘Expressed sequence tags’ (ESTs) have been collected by Sanger sequencing of individual cDNA clones isolated from a range of cells and tissues. Alignment of ESTs deposited in Genbank^28^ to the human genome using blat^29^ identifies all those containing a canonical intron and therefore derived from a spliced transcript. Of 57 spliced ESTs representing the VEGFA gene which include exon 8, 48 contain the standard 8a splice site and there are none with the putative VEGFA_xxx_b exon 8b start site (Fig. 2). One EST, CN256175 represents a rare putative novel isoform similar to transcript variant 6 (NM_001025370; which encodes isoform f with a non-AUG (CUG) translation initiation codon) which is spliced from exon 5 to a position upstream of the usual exon 8a site. Another putative novel upstream site is shared by the remaining 8 ESTs. These are all from the same hepatocellular carcinoma library (LIBEST_006533)^30^ and may represent a tumour-related transcript not represented in the cognate library from corresponding non-cancerous liver tissue (LIBEST_005601).

### Searches of three publicly available NGS datasets using BLAST and the STAR aligner

It is conceivable that the VEGFA_xxx_b splice site might not be represented within the small number of human ESTs available (<50 containing the canonical exon 8a splice site). Fortunately the advent of NGS has led to the creation of vast repositories of publicly available RNA-Seq data. We analysed three independent datasets comprising sequences from multiple human tissues; (1) 16 tissues from the Illumina Body Map (E-MTAB-513; Bioproject PRJEB2445; Study ERP000546) (adipose, adrenal, brain, breast, colon, heart, kidney, leukocyte, liver, lung, lymph node, ovary prostate, skeletal muscle, testis, thyroid); (2) 27 different tissues from 95 individuals analysed as part of the Human Protein Atlas (www.proteinatlas.org)^31^ (ArrayExpress accession E-MTAB-1733; PRJEB4337; ERP003613)(same tissues as data set 1 with addition of appendix, bladder, bone marrow, duodenum, endometrium, esophagus, fallopian tube, gall bladder, pancreas, placenta, rectum, salivary gland, skin, small intestine, smooth muscle, spleen, stomach and tonsil, but without breast or leukocytes); and (3) Selected tissues that contain cells reported to have expression of VEGFA_xxx_b: Normal kidney (reported to be expressed in glomerular podocytes^26, 32, 33^); retina^34^; placenta^35^; adipose (macrophages, reported source of VEGFA_xxx_b in peripheral artery disease^9^); and retinal pigment epithelial (RPE) cells^36–38^. Details of the datasets are provided in Supplementary Dataset 1.

A BLAST database (Supplementary Table 1) was created containing sequences of all the following potential exon junctions which contain the exon 8a or 8b splice sites (VEGFA isoform(s) encoded shown in brackets); 4-8 (VEGFA_111_), 5-8 (VEGFA_121_), 6a-8 (VEGFA_145_), 7a-8 (VEGFA_148_); and 7b-8 (VEGFA_165_, VEGFA_183_, VEGFA_189_, VEGFA_206_). BLAST searches were performed with each NGS read.

BLAST searches of the 5 billion (5 × 10^9^) reads in dataset 1 identified 10,188 reads containing perfect matches to the canonical VEGFA exon 8a splice junction and none to the VEGFA exon 8b junction. Similarly, of a total of 6.9 billion (6.9 × 10^9^) individual reads (3.5 billion pairs) in dataset 2, 31,614 reads matched the canonical VEGFA exon 8a splice junction and none matched the VEGFA exon 8b junction. Of a total of 2.8 billion (2.8 × 10^9^) reads in dataset 3 there were 8,428 matching exon 8a and none matching 8b. Dataset 3 included 6 ribosome profiling samples from kidney^39^, in which 11 reads containing the VEGFA exon 8a splice site were identified, but none with the VEGFA exon 8b site. The results of the BLAST analyses are summarised in Table 1 and provided in full in Supplementary Dataset 2. One read containing the VEGFA_xxx_b splice site would have represented 0.002% of the VEGFA transcripts present across all the tissues examined; the failure to detect any suggests that the overall proportion of VEGFA exon 8b transcripts is less than this. It is possible that VEGFAxxxb transcripts are more abundant in an individual tissue, but with an average of 1807 reads containing a canonical VEGFA exon 8a splice junction in each of 14 tissues represented in both datasets 1 and 2, the proportion is unlikely to be >0.06% that one read would indicate.

**Table 1:** Summary of datasets used, BLAST exact 22-base hits and STAR unique alignments to VEGFA junctions 8a and 8b.

Dataset	Source	Num Reads	BLAST hits to VEGFA junctions		STAR alignments to VEGFA junctions	
			8a	8b	8a	8b
1	E-MTAB-513	5 × 10^9^	10,188	0	11,931	0 (24)
2	E-MTAB-2836	6.9 × 10^9^	31,614	0	34,131	0 (56)
3	adipose, kidney, placenta, macrophages, retina, RPE	1.6 × 10^9^	1,380	0	2,406	0 (2)

Paired reads are counted here as two separate reads. No reads supported existence of an 8b splice sites. Prior to removal of the two VEGFA_xxx_b transcripts from the ‘genes.gtf’ annotation file a small number of reads (indicated in brackets) were reported to align to the annotated 8b splice site but these had very limited overlap in exon 7 (see Supplementary Fig. 1).

**Figure 3:**
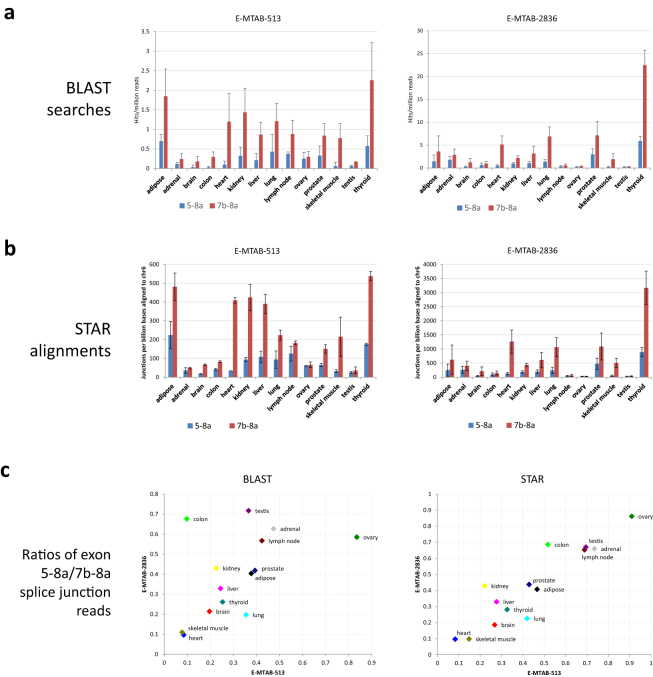
Occurrence of exon 8 splice junction sequences in RNA-Seq data from multiple tissues. Two multi-tissue RNA-Seq datasets (E-MTAB-513 and E-MTAB-2836) were interrogated for the presence of VEGFA exon 8 splice junctions. The number of reads reflecting splicing from exons 4, 6a and 7a were relatively few compared to those for the principal 5-8a and 7b-8a junctions (Supplementary Datasets 2 and 3). No sequences supporting the existence of the 8b site were detected. (**a**) The numbers of reads identified by BLAST searches to contain exon 5-8a and 7b-8a junction sequences are depicted across 14 tissues common to both datasets. (**b**) The number of exon 5-8a and 7b-8a splice junctions detected per billion bases aligned using the STAR aligner across the 14 common tissues. (**c**) The ratios of reads reflecting exon 5-8a: exon 7b-8a splice junctions for each tissue are plotted against the same tissue in the independent dataset. Both BLAST analysis (left) or STAR alignment (right) demonstrate correlation, with R^2^ values of 0.26 and 0.83 respectively. This demonstrates that specific tissues have characteristic VEGFA splicing patterns, with the abundance of transcripts containing exon 5-8a splicing relative to those with 7b-8a splicing ranging from 0.1–0.9.

An analysis was performed to reveal the tissue distribution of isoforms across the 14 tissues represented in both datasets and including several with reported high expression of VEGFA_xxx_b isoforms (eg kidney^8^ and adipose^40^). The vast majority (>99%) of all reads including an exon 8 splice junction in the 14 tissues common to both datasets included either the exon 5-8a junction characteristic of VEGFA_121_ or the exon 7b-8a junction present in multiple VEGFA isoforms. No reads containing any of the putative VEGFA_xxx_b junctions were detected. The numbers of hits per million reads to each splice junction are shown in Fig. 3a (data for all tissues listed in Supplementary Dataset 2). Fewer reads were detected for the exon 5-8a junction than the exon 7b-8a junction, with the proportion for each tissue generally being consistent between the two independent datasets (R^2^ = 0.26) and ranging from 0.1–0.8 (Fig. 3c).

Following the failure to identify VEGFA_xxx_b splice junctions by searching for the predicted sequences we employed the alternative strategy of aligning the reads to the hg19 genomic DNA reference using the Spliced Transcripts Alignment to a Reference (STAR) software^41^. Almost two million reads (1,994,279) aligned to the VEGFA region (chr6, from 43737946 to 43754223 on hg19), and of these there were 215,382 unique read alignments to splice events within the VEGFA gene. When the VEGFA_xxx_b transcripts were included in the guide gene.gtf file several reads did align to the VEGFA_xxx_b splice sites. However, on closer inspection these all had minimal overlap with exon 7 due to misalignment of the end three bases of the reads which are common to the end of exon 7 and the end of exon 8a immediately before the start of the proposed exon 8b splice site (see Supplementary Fig. 1 showing Integrated Genome Viewer (IGV) alignment). When the VEGFA_xxx_b transcripts (NM_001171629 and NM_001033756) were removed from the genes.gtf file, and the STAR alignment rerun with the novel splice option for the samples with putative VEGFAxxb sites, no reads aligned to the VEGFA_xxx_b splice site. The results of the STAR alignment to the VEGFA gene are summarised in Table 1 and listed in full in Supplementary Dataset 3. As observed from BLAST searches, more reads aligned to the exon 5-8a junction than to the exon 7b-8a junction (Fig. 3b), with ratios ranging from 0.1 (heart and skeletal muscle) to 0.9 (ovary). The values for the same tissue from the independent datasets were strongly correlated (R^2^ = 0.83) (Fig. 3c). The greater correlation between the same tissues observed with STAR alignments than with BLAST searches probably reflects the more effective normalisation possible with this approach (to bases aligned to chromosome 6) and the requirement for an exact match across the whole junction sequence with BLAST.

### Analysis of Genotype-Tissue Expression (GTEx) Tophat alignments

As part of the Genotype-Tissue Expression (GTEx)^24, 25^ project RNA-Seq reads from approximately 7000 samples from 50 tissues (Release V6) have been mapped to the human genome using Tophat). Whilst the relative tissue expression of exon 8a-containing VEGFA transcripts is in good agreement with our analyses of the Illumina Body Map and Human Protein Atlas data, with thyroid having the highest expression in all datasets (Fig. 3 and Supplementary Fig. 2), no reads are reported for either of the ENSEMBL transcripts containing the exon 8b splice site (ENST00000482630 and ENST00000518824).

### Sequencing of RT-PCR products spanning exon 7-8 junction

**Figure 4:**
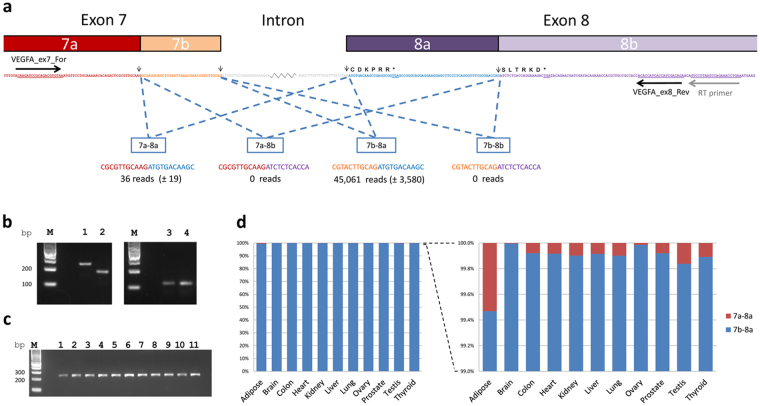
RT-PCR spanning VEGFA exons 7 to 8 and sequencing of amplification products. (**a**) The potential splice junctions between VEGFA exons 7 and 8 are indicated diagrammatically. The mean numbers of reads across all tissues (excluding adipose) containing each splice junction in RT-PCR products amplified using the primers shown in exons 7 and 8 are indicated. (**b**) Single PCR products of the sizes predicted for VEGFA_165_ (exon 7b-8a; 235bp) or VEGFA_165_b (exon 7b-8b; 169bp) were amplified from cells overexpressing the respective transcripts. Lane 1: HEK293 expressing VEGFA_165_, Lane 2: HEK293 expressing VEGFA_165_b, Lane M: Size marker. Products of the same size (115bp) were amplified using primers spanning exons 4 to 5 and common to both transcripts. Lane 3: HEK293 expressing VEGFA_165_, Lane 4: HEK293 expressing VEGFA_165b_. (**c**) The only RT-PCR products amplified by primers in exons 7 and 8 in all tissues and observed by agarose gel electrophoresis are consistent with amplification of exon 7b-8a transcripts (235bp). Lane 1: Adipose, Lane 2: Brain, Lane 3: Colon, Lane 4: Heart, Lane 5: Kidney, Lane 6: Liver, Lane 7: Lung, Lane 8: Ovary, Lane 9: Prostate, Lane 10: Testis, Lane 11: Thryoid, Lane M: Size marker. (**d**) Percentage of reads from transcripts with 7a-8a and 7b-8a splice events.

To assess the splicing between exons 7 and 8 with greater sensitivity, total RNA from a range of tissues and HEK293 cells expressing recombinant VEGFA_165_ or VEGFA_165_b transcripts was reverse transcribed from an oligonucleotide specific for exon 8 and PCR then performed with a primer pair located further upstream in exon 8 and in exon 7 (Fig. 4a). Single products of the sizes predicted for VEGFA_165_ (exon 7b-8a) or VEGFA_165_b (exon 7b-8b) were amplified from cells overexpressing the respective transcripts (Fig. 4b). From all tissues a single product consistent with exon 7b-8a was observed following agarose gel electrophoresis (Fig. 4c). However, use of next generation sequencing to determine the sequences of approximately 50,000 products from each PCR reaction revealed that while the majority (>99%) of reads were of the canonical exon 7b-8a splice site present in VEGFA_165_, VEGFA_183,_ VEGFA_189_, and VEGFA_206_ a small number were of the shortened exon 7, 7a-8a splice site reported in VEGFA_148_^26^ (Fig. 4d). No sequences of splicing events including exon 8b were detected in any of the eleven tissues tested.

### RNA-Seq analysis of cells expressing recombinant VEGFA_165_ or VEGFA_165_b transcripts

RNA from HEK293 cells expressing either recombinant VEGFA_165_ or VEGFA_165_b transcripts was subjected to RNA sequencing. Alignment of the resulting reads to the genome detected the expected 7b-8a or 7b-8b splice sites respectively. A coverage of approximately 20,000 reads along the length of each of the recombinant VEGF transcripts confirms that when over-expressed, both splice variants are detected with similar efficiency by RNA sequencing (Supplementary Fig. 3).

### Novel splice sites

**Figure 5:**
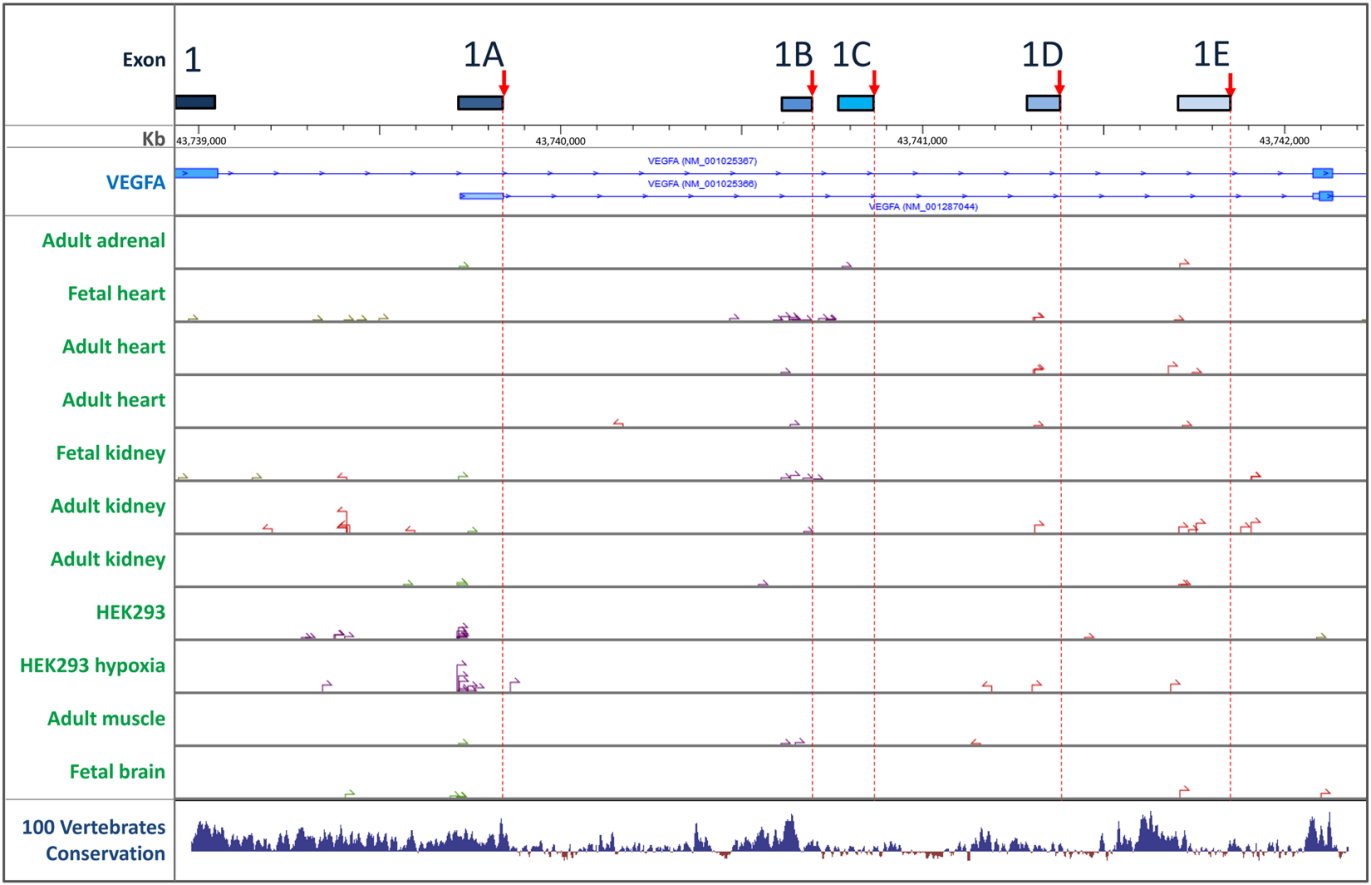
Novel exon 1 splice sites and predicted transcription start sites. Positions of novel splice sites are indicated by red arrows and dotted red lines and transcription start sites (TSS) from selected tissues by arrows.

Further analysis of the STAR alignments of the public datasets to the VEGFA gene identified numerous novel splice sites, in many cases supported by more reads than several previously annotated splice sites (Supplementary Dataset 3). One novel splice site is within exon 1 and is most frequently spliced to exon 2, creating a shortened exon 1 we have termed exon 1a (Fig. 1b). This site is also supported by several reads in which it is spliced to exon 3 and eight reads in which it is spliced to downstream within exon 1 creating a short intron and a transcript with CDS predicted to start at the canonical ATG. Of the novel splice junctions supported by more than 20 uniquely mapped reads four are between putative novel first exons and exon 2. The existence of the putative novel first exons is supported by the presence of transcript start sites just upstream of the predicted splice sites (Fig. 5). These were determined by Transcriptional Start Site-Sequencing (TSS-Seq), which uses oligo-capping to determine transcript 5′ ends^42^ and are reported in the DataBase of Transcriptional Start Sites (DBTSS: http://dbtss.hgc.jp/). The exon 1a splice site is before the first potential start codon and the putative novel first exons are likely to be non-coding (they either have no ATG codon or ones located very near the beginning of the exon which create ORFs differing from the canonical VEGFA peptide in subsequent exons and leading to premature termination). The CDS of these transcripts is therefore likely to start in exon 2, with the encoded VEGFA isoforms having a shorter N-terminus lacking a signal peptide and presumably retained within the cell.

**Figure 6:**
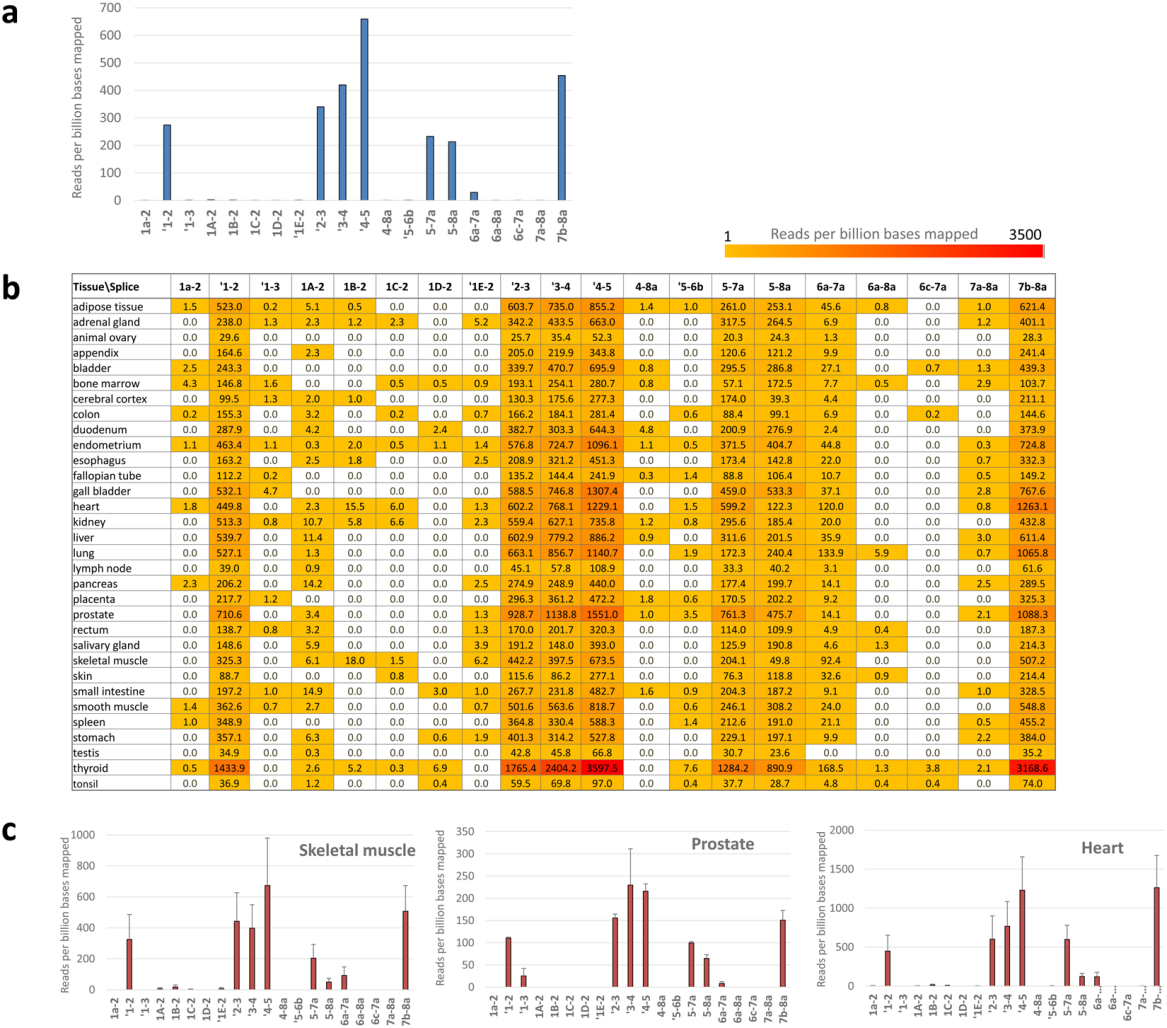
Occurrence of novel splicing events across different tissues. (**a**) All splice sites supported by 20–200 uniquely aligned reads from all datasets combined are listed and the number of reads per million aligned to each indicated. (**b**) The pattern of splice site use across different tissues (dataset 2) is indicated by the number of reads containing each site per billion bases mapped. (**c**) Examples of differential splice site use across tissues.

Multiple reads supporting the novel exon 1B-2 splicing event were detected in heart and kidney (Supplementary Dataset 3) and are matched by several potential cognate TSS’s in adult heart and kidney and many more in foetal heart and kidney^43^ (Fig. 5). Other novel splice junctions were detected between exons 1 and 3 and between exon 5 and a novel site within exon 6, 8 nucleotides downstream from the canonical 5′ site, although both events alter the reading frame and are predicted to cause premature termination of translation. The novel splicing events have been incorporated into an updated model of VEGFA splicing (Fig. 1b). The novel rare splice variants do not correlate with overall expression of VEGFA (Supplementary Fig. 4) and therefore are unlikely to reflect ‘mis-splicing’ of a fixed small fraction of all transcripts. The tissue specificity of the novel splicing events, together with previously reported splice junctions within the same range of expression, is illustrated in Fig. 6.

## Discussion

The use of alternative splicing to generate VEGFA protein isoforms with varying bioavailability is a key mechanism in the control of vascular development and function. The suggestion that additional alternative splicing to generate antiangiogenic VEGFA_xxx_b isoforms provides a further layer of regulation for this pivotal growth factor is therefore very plausible. However the analyses of RNA sequencing data presented in this study suggest that VEGFA_xxx_b transcripts are present at extremely low concentrations or do not exist *in vivo*. It seems unlikely that the absence of reads spanning the putative exon 8b junction is due to technical reasons associated with the laboratory procedures. Although all sequences are not represented equally by RNA-Seq reads due to the influences of factors such as ligation bias^44^ and varying reverse transcription efficiency, reads spanning exon 8b would be derived from RNAs with varying GC contents and a wide range of secondary structures, particularly if spliced to different upstream exons; therefore at least some would be amenable to sequencing. The detection using RNA-Seq of a similar number of reads spanning the exon 7b-8a and exon 7b-8b splice junctions from recombinant VEGF_165_ and VEGFA_165b_ constructs respectively confirms that both variants are detectable with similar efficiency using this technology. The exon 8a and exon 8b splice sites were also detected with comparable efficiency by RT-PCR, further confirming a lack of stable secondary structures which might block reverse transcription. Other factors which might cause mild under-representation of certain sequences differ between protocols and yet no VEGFA_xxx_b reads were observed in libraries prepared from multiple samples and in different laboratories. Even in tissues reported to have high levels of VEGFA_xxx_b protein (up to 45% of total VEGFA protein) such as kidney^33^ and placenta^35^, no exon 8b splice sites were detected.

The use of two complementary approaches, BLAST searches for predefined short exon junction sequences and STAR spliced alignment of reads to genome sequence, provide high confidence that exon 8b-spanning reads were not missed during the bioinformatic analyses. The value of this dual approach was demonstrated by the observation of multimapping reads mapping to the shared 3 bp sequence at the end of exon 7 and exon 8a (when the putative exon 8b splice site was provided in the STAR index guide file). Although this was detected as an artefact due to the lack of reads with longer overlap, the issue did not arise in the BLAST analysis. Furthermore, a different aligner (TopHat) was used in the analysis of the GTEX data and no VEGFA_xxx_b-containing reads were identified. It is possible that VEGFA_xxx_b isoforms are expressed at levels below the limit of detection of the available RNA-Seq datasets. However, the depth provided by the combined RT-PCR and sequencing approach enabled robust detection of the rare (<0.1%) exon 7a-exon 8 splicing event characteristic of VEGFA_148_, but still did not identify exon 8b splicing. If VEGFA_165_b does exist below this level of detection its biological significance is most likely limited.

A re-evaluation of the literature is required in light of these findings, which suggest that VEGFA_xxx_b transcripts do not exist *in vivo*. Firstly, antibodies specific for the VEGFA_xxx_b C-terminal peptide have detected antigen across a range of tissues^11, 20, 45, 46^. Secondly, a number of studies have suggested that VEGFA_165_b has anti-angiogenic properties. Recently overexpression of VEGFA_165_b in adipose issue has been linked with impaired angiogenesis^40^. Although VEGFA was highly expressed in adipose tissue according to RNA-Seq data, we detected no sequences specific for VEGFA_165_b transcripts. Similarly it has been reported that VEGFA_165_b is upregulated in peripheral artery disease^9^. Overexpression of any truncated or modified protein might cause dominant negative effects upon the endogenous protein, and this could be the explanation for the observed anti-angiogenic effects of VEGFA_165_b. However, the demonstration by Eswarappa, S. M. *et al*.^21^ that read-through of the VEGFA stop codon leads to translation of an extended protein (VEGF-Ax) with the same C-terminus as the putative VEGFA_xxx_b isoforms provides an alternative explanation. VEGF-Ax could be responsible for the signal observed with antibodies raised against the VEGFA_xxx_b terminal peptide^21^. VEGF-Ax has been shown to be antiangiogenic^20, 21^ and VEGFA_xxx_b may exert its anti-angiogenic effects through a similar mechanism, indeed both proteins are defective in binding to neuropilin-1^21, 47^.

Use of antibodies against the C-terminal peptide encoded by VEGFA exon 8a has been suggested as an isoform-specific therapy that would not target anti-angiogenic VEGFA_xxx_b isoforms. Although likely effective it would now be more pertinent to confirm lack of reactivity with VEGF-Ax than VEGFA_xxx_b. It has been reported that the serine arginine protein kinase (SRPK) inhibitor, SRPIN340, modulates splicing of the VEGFA pre-mRNA to reduce formation of VEGFA_165_ with a concomitant relative increase in VEGFA_165_b^48^. Similar modulation of splicing to exon 8b has been proposed as an approach to reduce neuropathic pain^49^. mRNA-Seq should be performed in these models to determine whether VEGFA_165_b is present.

Several novel putative first exons, each supported by more splice junction reads than were observed for the previously characterised VEGFA_148_ were identified. Although relatively rare, these variants exhibited tissue-specific expression and may be highly expressed in a small subset of cells. The exquisite expression patterns could be regulated through specific promoters which are active only in particular cell types. The function of the encoded proteins, which lack the N-terminal region present in canonical VEGFA proteins, is unclear but could be significant for such cells.

The existence and anti-angiogenic properties of VEGFA_xxx_b protein isoforms with an alternative C-terminus have been widely reported. The lack of RNA-Seq evidence for the cognate transcripts within approximately fifteen billion (14.7 × 10^9^) reads suggests that they do not exist or are present at extremely low concentrations *in vivo*. However, many of these observations can be attributed to the presence of VEGF-Ax proteins which have the same C terminus due to programmed translational readthrough rather than alternative splicing. The physiological implications of anti-angiogenic VEGFA isoforms and potential therapeutic applications therefore remain valid, but with the focus shifting to VEGF-Ax. The characterisation of additional VEGFA splice variants from RNA-Seq data, notably novel first exons, underlines the role of alternative promoters and splicing in delivering the exquisite regulation required for this pivotal gene.

## Methods

### Nomenclature

VEGFA exons are referred to as depicted in Fig. 1a and the co-ordinates in human reference hg19 are: Exon 1a 43737946–43738362; 1b 43738363–43739049; 1A 43739722–43739838; 1B? –43740695; 1C? –43740870; 1D?– 43741375; 2 43742078–43742129; 3 43745206–43745402; 4 43746197–43746273; 5 43746626–43746655; 6a 43748469–43748522; 6b 43748523–43748540; 6c 43748541–43748591; 7a 43749693–43749789; 7b 43749790–43749824; 8a 43752278–43752343; 8b 43752344–43754223.

Splice junctions were named according to the donor and acceptor exons. Junction 7b-8a is present in transcripts encoding the VEGFA_165_, VEGFA_183,_ VEGFA_189_ and VEGFA_206_ isoforms. Junction 5-8a is present in transcripts encoding the VEGFA_121_ isoform.

### Datasets

RNA-Seq datasets were identified in Gene Expression Omnibus (GEO)^50^ (http://www.ncbi.nlm.nih.gov/geo/) or ArrayExpress (http://www.ebi.ac.uk/arrayexpress/). The two principal datasets comprised: 16 human tissues from the Illumina bodyMap2 transcriptome (http://www.ncbi.nlm.nih.gov/bioproject/204271) (dataset 1) and tissue samples of 95 human individuals representing 27 different tissues^31^ (dataset 2). Dataset 3 contained additional RNA sequencing datasets from a range of studies from kidney, retina, placenta, adipose tissue, macrophages and RPE^36–38^. These 3 datasets are summarised in Table 1 and in more detail in Supplementary Dataset 1. Data for the VEGFA gene was downloaded from the GTEx portal (http://www.gtexportal.org/home/gene/VEGFA). Expressed sequence tags (ESTs) with evidence of splicing were viewed using the UCSC Genome browser (http://genome-euro.ucsc.edu). Transcription start sites were identified from the Database of Transcriptional Start Sites (DBTSS: http://dbtss.hgc.jp/), an integrative platform for transcriptome, epigenome and genome sequence variation data^51^.

### Data analysis

#### Blastn search for splice sites

A fasta file of all ten VEGFA splice junctions including exon 8a or 8b was created with each sequence of length 22 bases (ie. 11 bases either side of the splice site), see Supplementary data Table 1). This was indexed using the “*formatdb*” command from a locally installed copy of the NCBI blastall version 2.2.26 (NCBI *blastall* – ftp://ftp.ncbi.nlm.nih.gov/blast/executables/blast/LATEST/)^52^. Fastq files of Illumina RNAseq reads (as listed in Supplementary dataset 1), were downloaded from the EBI’s sequence read archive: 16 human tissues, Illumina bodyMap2 transcriptome: http://www.ncbi.nlm.nih.gov/bioproject/204271) and RNA-seq of coding RNA from tissue samples of 95 human individuals representing 27 different tissues^31^. These fastq files of reads were converted to fasta format using a perl script. *Blastn* (from the locally installed NCBI *blastall*) was used to search the exon junctions file (described above) for matches to these reads that had an e-value cutoff 1e-6 or better, with complexity filtering disabled (ie. *blastall* options: *-p blastn -e 1e-6 -F F -m 8*). Read 1 and read 2 from the paired-end reads were used independently. To compare between the different runs and tissues, the numbers of hits were normalised to reads per million in each run. A perl script was used to summarise the results from all runs as a text file which was subsequently imported into Excel for viewing and plotting.

#### Splice-aware alignment to human reference

The STAR RNAseq aligner (version 2.4.0f1)^41^ was used to align the Illumina reads (obtained as in point 1 above) to the human reference hg19 chromosome 6, using options:

STAR–runMode alignReads–genomeDir $HG19chr6–runThreadN 4–genomeLoad LoadAndRemove–readFilesIn $IN1.fastq $IN2.fastq–readFilesCommand zcat–outSAMmode Full–outSAMattributes Standard–outFileNamePrefix $OUT.

A Perl script (available on request) was written to extract the splice sites within the VEGFA gene (nucleotide position 43737945 to 43754224 on chromosome 6 of the hg19 genome) from all the splice-junction files produced by STAR, summarising these in text files subsequently imported into Excel (see Supplementary Dataset 3) for viewing and plotting. To compare between the different runs and tissues, numbers of VEGFA splice sites were normalised as ‘per billion bases aligned to chromosome 6’. Using samtools^53^, the bam files of STAR alignments were sorted by position and indexed, to view the alignments in the IGV browser^54^.

### Generation of recombinant VEGF expression plasmids

Sequences encoding the VEGFA_165_ or VEGFA_165_b open reading frames and 205 nucleotides of the 3′ UTR were synthesised and inserted into pcDNA3.1(+)(Thermo Fisher) at the *Hin*dIII/*Eco*RI restriction sites (see Supplementary Dataset 4). The plasmid DNA was purified from transformed bacteria (*E. coli* K12 DH10B™ T1R) and concentration determined by UV spectroscopy. The final construct was verified by Sanger sequencing, confirming 100% sequence congruence within the insertion sites.

### Cell culture and RNA extraction

HEK293 cells were cultured in DMEM (Thermo Fisher) supplemented with 10% fetal calf serum (FCS) and 100 μg/ml PrimocinTM (InvivoGen). Transfections were performed with 1 μg plasmid using lipofectamine 3000 (Thermo Fisher) and following the manufacturer’s instructions for a 12 well plate. RNA extractions were performed after 24 hours using a miRCURY RNA Isolation Kit (Exiqon) and following the manufacturer’s instructions.

### RT-PCR and NGS

Total human RNA (FirstChoice Human Total RNA Survey Panel, Life Technologies) (1 µg) or RNA from HEK293 cells was reverse transcribed at 50 °C using an oligonucleotide (TTCAGGTTTCTGGATTAAGGAC) specific for VEGFA exon 8 (Fig. 4) and Superscript III (Life Technologies). PCR was performed with primers spanning the exon7-8 splice junction (VEGFA_ex7_For1 CAAGATCCGCAGACGTGTAA; VEGFA_ex8_Rev TCTGTCGATGGTGATGGTGT) or within exons 4 and 5 of VEGFA_165_ and VEGFA_165_b (VEGF_universal_For TGCGGATCAAACCTCACCAA; VEGF_universal_Rev GGCCCACAGGGATTTTCTTG). PCR amplification was performed using Platinum PCR SuperMix High Fidelity Taq (Life Technologies) with an initial denaturation at 95 °C for 3 min, followed by 40 cycles of 95 °C for 30 s, 55 °C for 30 s and 68 °C for 1 min. Amplicon size was verified by gel electrophoresis on a 2% agarose gel.

Libraries were prepared from PCR products using the Ion Plus fragment library kit (Life Technologies) following the manufacturer’s instructions (MAN0006846) version A.0 with slight modifications. Pooling was not performed until after the libraries were barcoded using the Ion express barcode adaptor kit (Life Technologies). All purification steps were performed using Agencourt AMPure XP Reagent (Beckman Coulter) as indicated in the protocol. Libraries were quantified using the PerfeCTa NGS Quantification Kit for Ion Torrent libraries (Quanta Biosciences). Template preparation was performed on the OneTouch 2 system (Life Technologies) using the Ion PGM Template OT2 200 Kit and protocol version A.0 as recommended by the manufacturer’s instructions (Life Technologies). Sequencing was performed on Ion Torrent PGM (Life Technologies) using the Ion PGM HI-Q Sequencing kit and an Ion 314 Chip Kit V2, following the manufacturer’s instructions (version B.0).

TruSeq stranded mRNA-seq libraries were prepared from total RNA by Edinburgh Genomics, The University of Edinburgh and sequenced on a MiSeq (v2 100PE).

## Electronic supplementary material

Supplementary Information

Supplementary Suppl Dataset 1

Supplementary Suppl Dataset 2

Supplementary Suppl Dataset 3

Supplementary Suppl Dataset 3
