# Supplementary material for: RNA-Sequencing data supports the existence of novel VEGFA splicing events but not of VEGFAxxxb isoforms
Source: Sci Rep. 2017 Mar 3;7:58. doi: 10.1038/s41598-017-00100-3 (PMC5427905; doi:10.1038/s41598-017-00100-3)
Supplement: Supplementary file 1 — Supplementary Information [file 41598_2017_100_MOESM1_ESM.pdf]

Supplementary information

RNA-Sequencing data supports the existence of novel VEGFA splicing events but not of VEGFAxxxb isoforms

Stephen Bridgett†, Margaret Dellett† and David A. Simpson*

*Address correspondence to David Arthur Simpson: Centre for Experimental Medicine, The Wellcome – Wolfson Institute for Experimental Medicine, School of Medicine, Dentistry and Biomedical Sciences, Queen's University Belfast, 97 Lisburn Road, Belfast, BT9 7BL, Northern Ireland, United Kingdom. E-mail: David.Simpson@qub.ac.uk
†these authors contributed equally

Supplementary Figure 1. IGV browser showing misalignment of reads to the putative VEGFA exon 8b splice site. The 3 nucleotides (CAG) upstream of the exon 7 splice junction are the same as those upstream of the putative exon 8b site. Therefore reads that begin just upstream of the annotated 8b site can be misinterpreted as having been spliced from exon 7. If transcripts with exon 7-8b splicing existed, junction-spanning reads with more sequence within exon 7 would be observed.

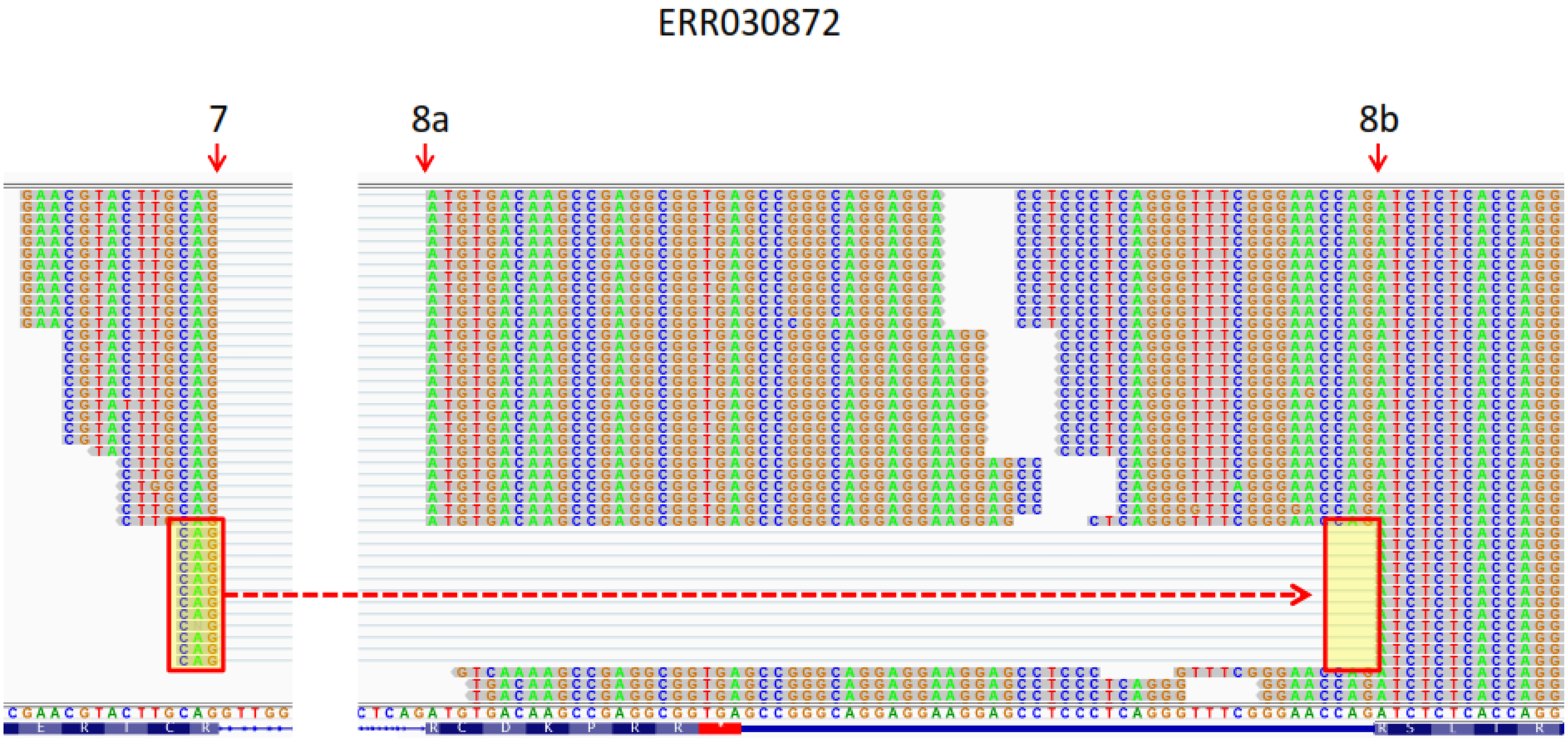

Supplementary Figure 2. Splicing of the VEGFA gene reported by the Genotype-Tissue Expression project (GTEx)[24,25]. The first five tissues ranked by VEGFA expression are shown. A median of 1046.5 read counts is reported for the exon 7-8a junction in thyroid. Although a median read count of 1 is reported for the exon7-8b junction in thyroid, this seems likely to be an alignment error because no reads are reported in the other tissues and no reads are recorded for either of the Ensembl transcripts containing the putative exon7-8b junction (ENST00000482630 & ENST00000518824).

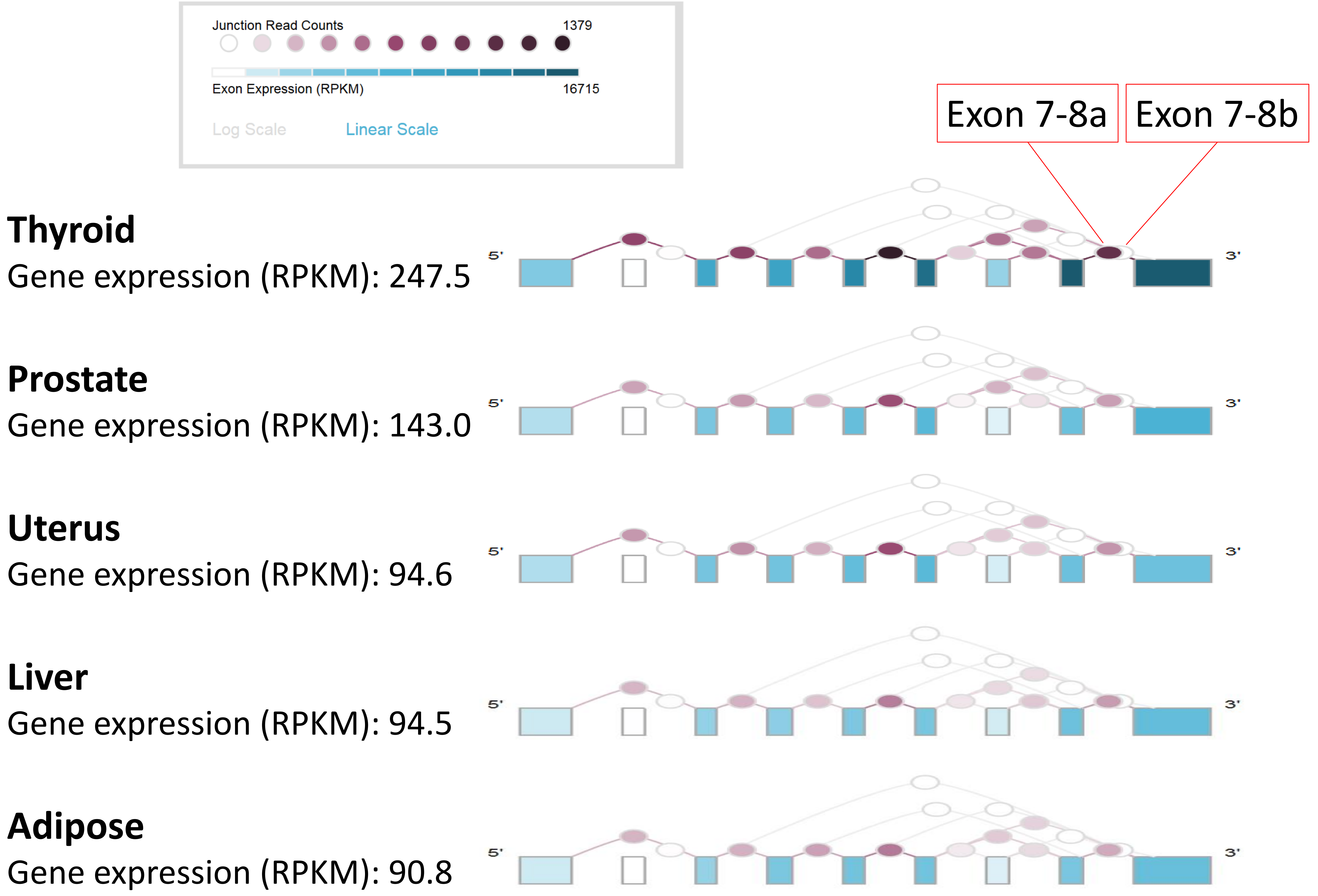

Supplementary Figure 3. Alignment of RNA-Seq reads from cells expressing either recombinant VEGFA$_{165}$b or VEGFA$_{165}$ to exon 8 of the VEGFA gene. The number of reads detected from cells expressing VEGFA$_{165}$b which spanned the exon 7b-8b splice site was comparable to those along the rest of the transcript (~20,000) (top panel). Likewise, for cells expressing VEGF$_{165}$ a similar number of reads were detected for the whole transcript, including the exon7b-8a splice site. The total coverage (top) and representative reads are shown displayed in the IGV browser.

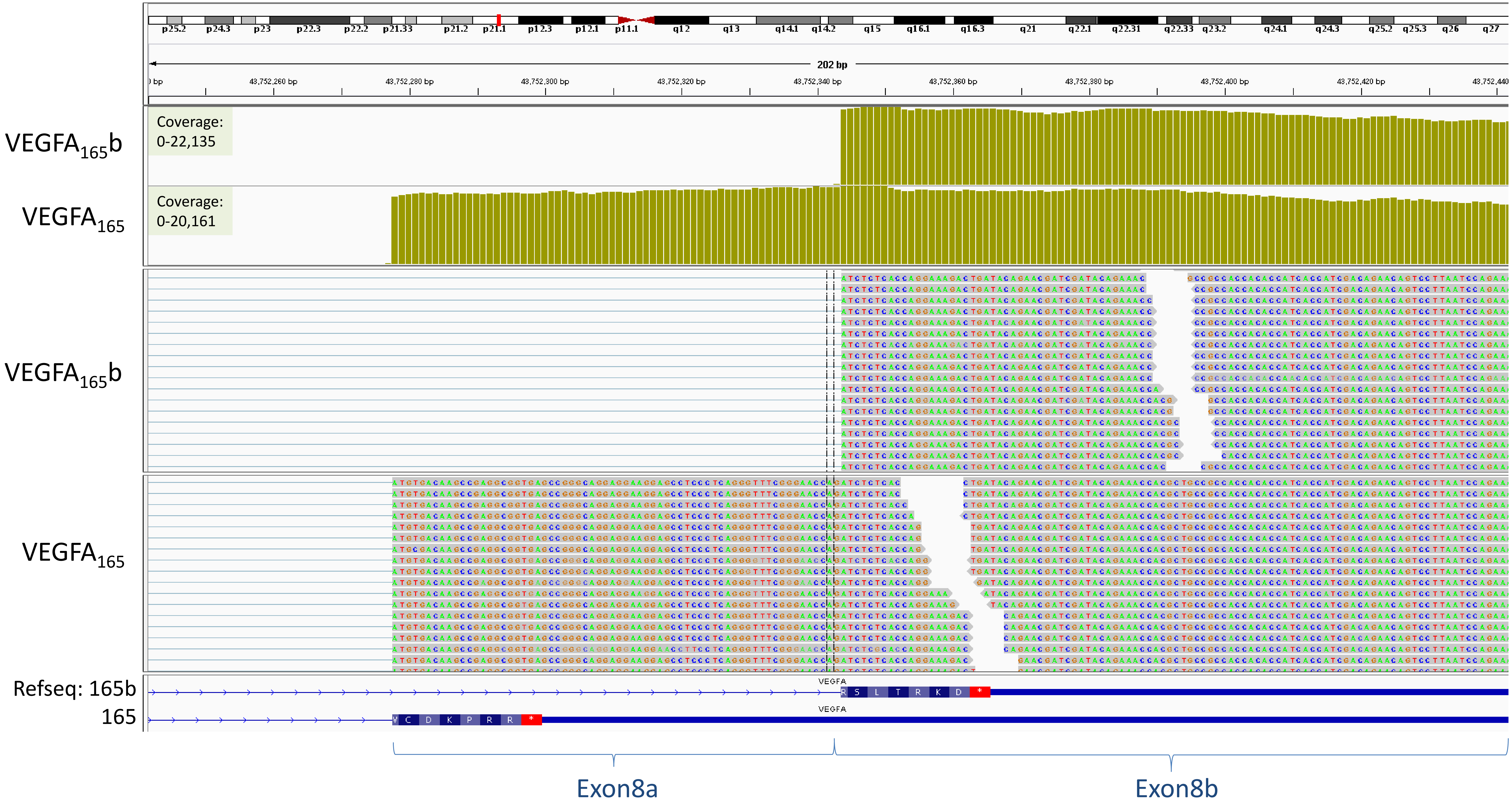

Supplementary Figure 4. Relative abundance of VEGFA splice junctions. Tissues are arranged by total abundance of VEGFA splice site reads. The numbers of reads spanning the most abundant exon 5-8 and exon 7b-8 splice sites decrease broadly in line with total VEGFA reads (A). In contrast the expression of rare variants is largely independent of overall VEGFA expression (B).

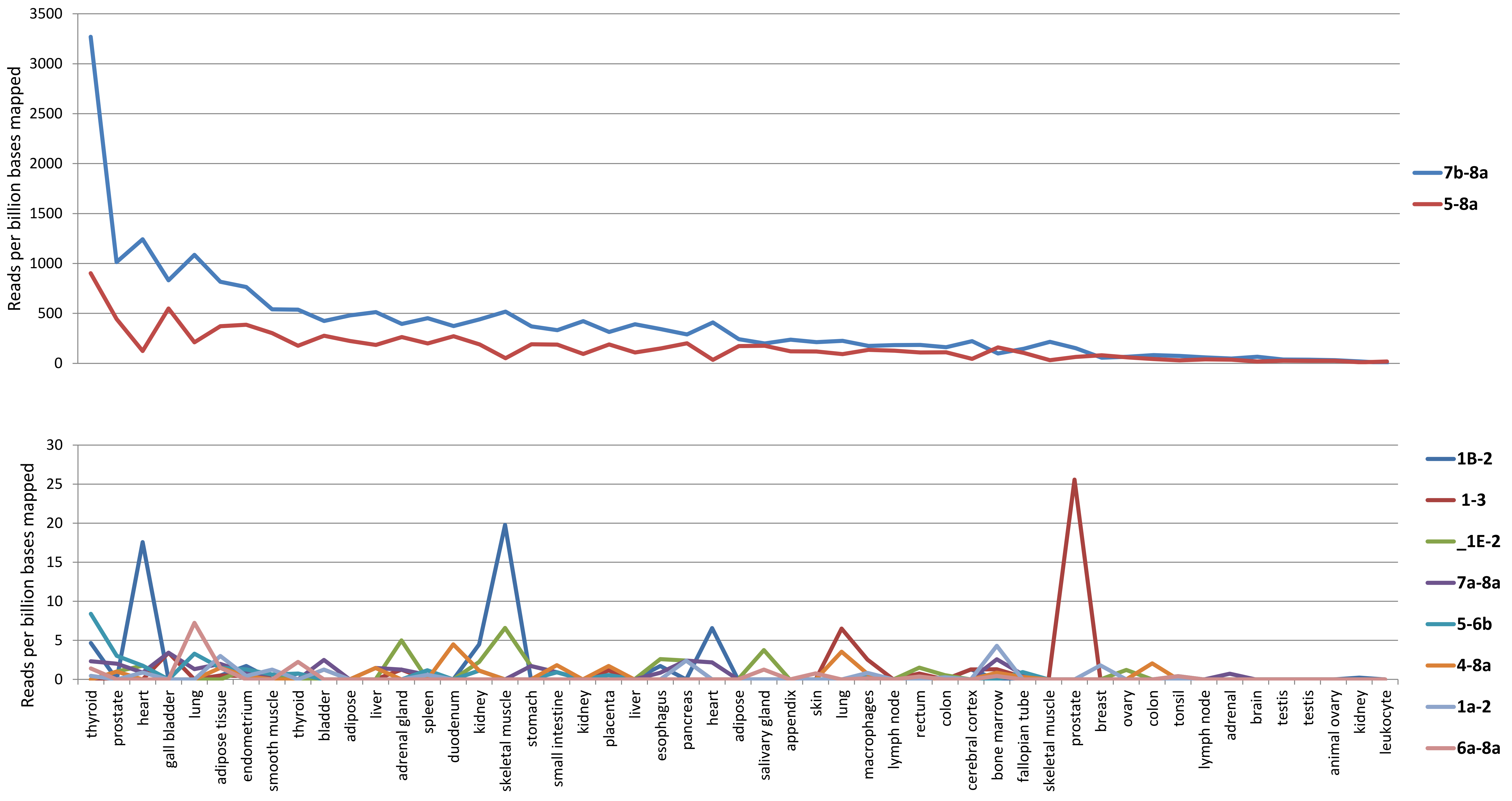

Supplementary Table 1. Sequences of exon junctions containing exon 8 splice sites used for BLAST searches (fasta format).

```
>VEGFA exon4_8a_splice_junction gi|284172472
TGTGAATGCAGATGTGACAAGC
>VEGFA exon4_8b_splice_junction gi|284172472
TGTGAATGCAGATCTCTCACCA
>VEGFA  exon5_8a_splice_junction gi|284172468
AGACAAGAAAAATGTGACAAGC
>VEGFA exon5_8b_splice_junction gi|284172468
AGACAAGAAAAATCTCTCACCA
>VEGFA exon6a_8a_splice_junction gi|324120931
TCCTGGAGCGTATGTGACAAGC
>VEGFA exon6a_8b_splice_junction gi|324120931
TCCTGGAGCGTATCTCTCACCA
>VEGFA exon7a_8a_splice_junction gi|284172466
CGCGTTGCAAGATGTGACAAGC
>VEGFA exon7a_8b_splice_junction gi|284172466
CGCGTTGCAAGATCTCTCACCA
>VEGFA_exon7b_8a_splice_junction
CGTACTTGCAGATGTGACAAGC
>VEGFA_exon7b_8b_splice_junction
CGTACTTGCAGATCTCTCACCA
```
