# Supplementary material for: RNA-Sequencing data supports the existence of novel VEGFA splicing events but not of VEGFAxxxb isoforms
Source: Sci Rep. 2017 Mar 3;7:58. doi: 10.1038/s41598-017-00100-3 (PMC5427905; doi:10.1038/s41598-017-00100-3)
Supplement: Supplementary file 5 — Supplementary Suppl Dataset 3 [file 41598_2017_100_MOESM5_ESM.doc]

| **Suppl Dataset 4: Fasta files of the VEGFA_165_ and VEGFA_165_b sequences which were cloned into pcDNA 3.1 and Genbank files of the recombinant plasmids.** |
| --- |

**VEGFA_165_**

>VEGFA165_orf_3UTR

Atgaactttctgctgtcttgggtgcattggagccttgccttgctgctctacctccaccatgccaagtggtcccaggctgcacccatggcagaaggaggagggcagaatcatcacgaagtggtgaagttcatggatgtctatcagcgcagctactgccatccaatcgagaccctggtggacatcttccaggagtaccctgatgagatcgagtacatcttcaagccatcctgtgtgcccctgatgcgatgcgggggctgctgcaatgacgagggcctggagtgtgtgcccactgaggagtccaacatcaccatgcagattatgcggatcaaacctcaccaaggccagcacataggagagatgagcttcctacagcacaacaaatgtgaatgcagaccaaagaaagatagagcaagacaagaaaatccctgtgggccttgctcagagcggagaaagcatttgtttgtacaagatccgcagacgtgtaaatgttcctgcaaaaacacagactcgcgttgcaaggcgaggcagcttgagttaaacgaacgtacttgcagatgtgacaagccgaggcggtgagccgggcaggaggaaggagcctccctcagggtttcgggaaccagatctctcaccaggaaagactgatacagaacgatcgatacagaaaccacgctgccgccaccacaccatcaccatcgacagaacagtccttaatccagaaacctgaaatgaaggaagaggagactctgcgcagagcactttgggtccggagggcgagactccggcggaagcattcccgggcgggtgacccagcacggtccctcttggaattggattcgccattttatttt

**VEGFA_165_b**

>VEGFA165b_orf_3UTR

Atgaactttctgctgtcttgggtgcattggagccttgccttgctgctctacctccaccatgccaagtggtcccaggctgcacccatggcagaaggaggagggcagaatcatcacgaagtggtgaagttcatggatgtctatcagcgcagctactgccatccaatcgagaccctggtggacatcttccaggagtaccctgatgagatcgagtacatcttcaagccatcctgtgtgcccctgatgcgatgcgggggctgctgcaatgacgagggcctggagtgtgtgcccactgaggagtccaacatcaccatgcagattatgcggatcaaacctcaccaaggccagcacataggagagatgagcttcctacagcacaacaaatgtgaatgcagaccaaagaaagatagagcaagacaagaaaatccctgtgggccttgctcagagcggagaaagcatttgtttgtacaagatccgcagacgtgtaaatgttcctgcaaaaacacagactcgcgttgcaaggcgaggcagcttgagttaaacgaacgtacttgcagatctctcaccaggaaagactgatacagaacgatcgatacagaaaccacgctgccgccaccacaccatcaccatcgacagaacagtccttaatccagaaacctgaaatgaaggaagaggagactctgcgcagagcactttgggtccggagggcgagactccggcggaagcattcccgggcgggtgacccagcacggtccctcttggaattggattcgccattttatttt

LOCUS VEGFA165_orf_3UTR_pcDNA3.1(+) 6244 bp DNA circular 31-JUL-2014

FEATURES Location/Qualifiers

promoter 863..881

/label="T7_promoter"

promoter complement(6150..6178)

/label="AmpR_promoter"

promoter 2686..2888

/label="SV40_promoter"

terminator 1838..2065

/label="bGH_PA_terminator"

CDS complement(5248..6108)

/label="Amp(R)"

CDS 2940..3740

/label="Neo(R)"

rep_origin complement(4474..5093)

/label="pBR322_origin"

rep_origin 2733..2810

/label="SV40_origin"

rep_origin 2128..2434

/label="f1_origin"

primer complement(4083..4101)

/label="M13_reverse_primer"

primer complement(1835..1852)

/label="BGH\rev\primer"

primer 769..789

/label="CMV_fwd_primer"

primer 4008..4027

/label="EBV_rev_primer"

primer complement(4100..4122)

/label="M13_pUC_rev_primer"

primer complement(2548..2568)

/label="pBABE_3_primer"

primer 2795..2814

/label="SV40pro_F_primer"

polyA_site 1841..2065

/label="BGH\pA"

promoter 308..818

/label="CMV_Promoter"

terminator 3922..4038

/label="SV40_PA_terminator"

promoter 236..852

/label="CMV_immearly_promoter"

CDS 2949..3743

/label="Neo_Kana"

CDS 911..1770

/label="VEGFA165_orf_part3UT"

ORIGIN

1 GACGGATCGG GAGATCTCCC GATCCCCTAT GGTGCACTCT CAGTACAATC TGCTCTGATG

61 CCGCATAGTT AAGCCAGTAT CTGCTCCCTG CTTGTGTGTT GGAGGTCGCT GAGTAGTGCG

121 CGAGCAAAAT TTAAGCTACA ACAAGGCAAG GCTTGACCGA CAATTGCATG AAGAATCTGC

181 TTAGGGTTAG GCGTTTTGCG CTGCTTCGCG ATGTACGGGC CAGATATACG CGTTGACATT

241 GATTATTGAC TAGTTATTAA TAGTAATCAA TTACGGGGTC ATTAGTTCAT AGCCCATATA

301 TGGAGTTCCG CGTTACATAA CTTACGGTAA ATGGCCCGCC TGGCTGACCG CCCAACGACC

361 CCCGCCCATT GACGTCAATA ATGACGTATG TTCCCATAGT AACGCCAATA GGGACTTTCC

421 ATTGACGTCA ATGGGTGGAG TATTTACGGT AAACTGCCCA CTTGGCAGTA CATCAAGTGT

481 ATCATATGCC AAGTACGCCC CCTATTGACG TCAATGACGG TAAATGGCCC GCCTGGCATT

541 ATGCCCAGTA CATGACCTTA TGGGACTTTC CTACTTGGCA GTACATCTAC GTATTAGTCA

601 TCGCTATTAC CATGGTGATG CGGTTTTGGC AGTACATCAA TGGGCGTGGA TAGCGGTTTG

661 ACTCACGGGG ATTTCCAAGT CTCCACCCCA TTGACGTCAA TGGGAGTTTG TTTTGGCACC

721 AAAATCAACG GGACTTTCCA AAATGTCGTA ACAACTCCGC CCCATTGACG CAAATGGGCG

781 GTAGGCGTGT ACGGTGGGAG GTCTATATAA GCAGAGCTCT CTGGCTAACT AGAGAACCCA

841 CTGCTTACTG GCTTATCGAA ATTAATACGA CTCACTATAG GGAGACCCAA GCTGGCTAGC

901 GTTTAAACTT AAGCTTATGA ACTTTCTGCT GTCTTGGGTG CATTGGAGCC TTGCCTTGCT

961 GCTCTACCTC CACCATGCCA AGTGGTCCCA GGCTGCACCC ATGGCAGAAG GAGGAGGGCA

1021 GAATCATCAC GAAGTGGTGA AGTTCATGGA TGTCTATCAG CGCAGCTACT GCCATCCAAT

1081 CGAGACCCTG GTGGACATCT TCCAGGAGTA CCCTGATGAG ATCGAGTACA TCTTCAAGCC

1141 ATCCTGTGTG CCCCTGATGC GATGCGGGGG CTGCTGCAAT GACGAGGGCC TGGAGTGTGT

1201 GCCCACTGAG GAGTCCAACA TCACCATGCA GATTATGCGG ATCAAACCTC ACCAAGGCCA

1261 GCACATAGGA GAGATGAGCT TCCTACAGCA CAACAAATGT GAATGCAGAC CAAAGAAAGA

1321 TAGAGCAAGA CAAGAAAATC CCTGTGGGCC TTGCTCAGAG CGGAGAAAGC ATTTGTTTGT

1381 ACAAGATCCG CAGACGTGTA AATGTTCCTG CAAAAACACA GACTCGCGTT GCAAGGCGAG

1441 GCAGCTTGAG TTAAACGAAC GTACTTGCAG ATGTGACAAG CCGAGGCGGT GAGCCGGGCA

1501 GGAGGAAGGA GCCTCCCTCA GGGTTTCGGG AACCAGATCT CTCACCAGGA AAGACTGATA

1561 CAGAACGATC GATACAGAAA CCACGCTGCC GCCACCACAC CATCACCATC GACAGAACAG

1621 TCCTTAATCC AGAAACCTGA AATGAAGGAA GAGGAGACTC TGCGCAGAGC ACTTTGGGTC

1681 CGGAGGGCGA GACTCCGGCG GAAGCATTCC CGGGCGGGTG ACCCAGCACG GTCCCTCTTG

1741 GAATTGGATT CGCCATTTTA TTTTGAATTC TGCAGATATC CAGCACAGTG GCGGCCGCTC

1801 GAGTCTAGAG GGCCCGTTTA AACCCGCTGA TCAGCCTCGA CTGTGCCTTC TAGTTGCCAG

1861 CCATCTGTTG TTTGCCCCTC CCCCGTGCCT TCCTTGACCC TGGAAGGTGC CACTCCCACT

1921 GTCCTTTCCT AATAAAATGA GGAAATTGCA TCGCATTGTC TGAGTAGGTG TCATTCTATT

1981 CTGGGGGGTG GGGTGGGGCA GGACAGCAAG GGGGAGGATT GGGAAGACAA TAGCAGGCAT

2041 GCTGGGGATG CGGTGGGCTC TATGGCTTCT GAGGCGGAAA GAACCAGCTG GGGCTCTAGG

2101 GGGTATCCCC ACGCGCCCTG TAGCGGCGCA TTAAGCGCGG CGGGTGTGGT GGTTACGCGC

2161 AGCGTGACCG CTACACTTGC CAGCGCCCTA GCGCCCGCTC CTTTCGCTTT CTTCCCTTCC

2221 TTTCTCGCCA CGTTCGCCGG CTTTCCCCGT CAAGCTCTAA ATCGGGGGCT CCCTTTAGGG

2281 TTCCGATTTA GTGCTTTACG GCACCTCGAC CCCAAAAAAC TTGATTAGGG TGATGGTTCA

2341 CGTAGTGGGC CATCGCCCTG ATAGACGGTT TTTCGCCCTT TGACGTTGGA GTCCACGTTC

2401 TTTAATAGTG GACTCTTGTT CCAAACTGGA ACAACACTCA ACCCTATCTC GGTCTATTCT

2461 TTTGATTTAT AAGGGATTTT GCCGATTTCG GCCTATTGGT TAAAAAATGA GCTGATTTAA

2521 CAAAAATTTA ACGCGAATTA ATTCTGTGGA ATGTGTGTCA GTTAGGGTGT GGAAAGTCCC

2581 CAGGCTCCCC AGCAGGCAGA AGTATGCAAA GCATGCATCT CAATTAGTCA GCAACCAGGT

2641 GTGGAAAGTC CCCAGGCTCC CCAGCAGGCA GAAGTATGCA AAGCATGCAT CTCAATTAGT

2701 CAGCAACCAT AGTCCCGCCC CTAACTCCGC CCATCCCGCC CCTAACTCCG CCCAGTTCCG

2761 CCCATTCTCC GCCCCATGGC TGACTAATTT TTTTTATTTA TGCAGAGGCC GAGGCCGCCT

2821 CTGCCTCTGA GCTATTCCAG AAGTAGTGAG GAGGCTTTTT TGGAGGCCTA GGCTTTTGCA

2881 AAAAGCTCCC GGGAGCTTGT ATATCCATTT TCGGATCTGA TCAAGAGACA GGATGAGGAT

2941 CGTTTCGCAT GATTGAACAA GATGGATTGC ACGCAGGTTC TCCGGCCGCT TGGGTGGAGA

3001 GGCTATTCGG CTATGACTGG GCACAACAGA CAATCGGCTG CTCTGATGCC GCCGTGTTCC

3061 GGCTGTCAGC GCAGGGGCGC CCGGTTCTTT TTGTCAAGAC CGACCTGTCC GGTGCCCTGA

3121 ATGAACTGCA GGACGAGGCA GCGCGGCTAT CGTGGCTGGC CACGACGGGC GTTCCTTGCG

3181 CAGCTGTGCT CGACGTTGTC ACTGAAGCGG GAAGGGACTG GCTGCTATTG GGCGAAGTGC

3241 CGGGGCAGGA TCTCCTGTCA TCTCACCTTG CTCCTGCCGA GAAAGTATCC ATCATGGCTG

3301 ATGCAATGCG GCGGCTGCAT ACGCTTGATC CGGCTACCTG CCCATTCGAC CACCAAGCGA

3361 AACATCGCAT CGAGCGAGCA CGTACTCGGA TGGAAGCCGG TCTTGTCGAT CAGGATGATC

3421 TGGACGAAGA GCATCAGGGG CTCGCGCCAG CCGAACTGTT CGCCAGGCTC AAGGCGCGCA

3481 TGCCCGACGG CGAGGATCTC GTCGTGACCC ATGGCGATGC CTGCTTGCCG AATATCATGG

3541 TGGAAAATGG CCGCTTTTCT GGATTCATCG ACTGTGGCCG GCTGGGTGTG GCGGACCGCT

3601 ATCAGGACAT AGCGTTGGCT ACCCGTGATA TTGCTGAAGA GCTTGGCGGC GAATGGGCTG

3661 ACCGCTTCCT CGTGCTTTAC GGTATCGCCG CTCCCGATTC GCAGCGCATC GCCTTCTATC

3721 GCCTTCTTGA CGAGTTCTTC TGAGCGGGAC TCTGGGGTTC GAAATGACCG ACCAAGCGAC

3781 GCCCAACCTG CCATCACGAG ATTTCGATTC CACCGCCGCC TTCTATGAAA GGTTGGGCTT

3841 CGGAATCGTT TTCCGGGACG CCGGCTGGAT GATCCTCCAG CGCGGGGATC TCATGCTGGA

3901 GTTCTTCGCC CACCCCAACT TGTTTATTGC AGCTTATAAT GGTTACAAAT AAAGCAATAG

3961 CATCACAAAT TTCACAAATA AAGCATTTTT TTCACTGCAT TCTAGTTGTG GTTTGTCCAA

4021 ACTCATCAAT GTATCTTATC ATGTCTGTAT ACCGTCGACC TCTAGCTAGA GCTTGGCGTA

4081 ATCATGGTCA TAGCTGTTTC CTGTGTGAAA TTGTTATCCG CTCACAATTC CACACAACAT

4141 ACGAGCCGGA AGCATAAAGT GTAAAGCCTG GGGTGCCTAA TGAGTGAGCT AACTCACATT

4201 AATTGCGTTG CGCTCACTGC CCGCTTTCCA GTCGGGAAAC CTGTCGTGCC AGCTGCATTA

4261 ATGAATCGGC CAACGCGCGG GGAGAGGCGG TTTGCGTATT GGGCGCTCTT CCGCTTCCTC

4321 GCTCACTGAC TCGCTGCGCT CGGTCGTTCG GCTGCGGCGA GCGGTATCAG CTCACTCAAA

4381 GGCGGTAATA CGGTTATCCA CAGAATCAGG GGATAACGCA GGAAAGAACA TGTGAGCAAA

4441 AGGCCAGCAA AAGGCCAGGA ACCGTAAAAA GGCCGCGTTG CTGGCGTTTT TCCATAGGCT

4501 CCGCCCCCCT GACGAGCATC ACAAAAATCG ACGCTCAAGT CAGAGGTGGC GAAACCCGAC

4561 AGGACTATAA AGATACCAGG CGTTTCCCCC TGGAAGCTCC CTCGTGCGCT CTCCTGTTCC

4621 GACCCTGCCG CTTACCGGAT ACCTGTCCGC CTTTCTCCCT TCGGGAAGCG TGGCGCTTTC

4681 TCATAGCTCA CGCTGTAGGT ATCTCAGTTC GGTGTAGGTC GTTCGCTCCA AGCTGGGCTG

4741 TGTGCACGAA CCCCCCGTTC AGCCCGACCG CTGCGCCTTA TCCGGTAACT ATCGTCTTGA

4801 GTCCAACCCG GTAAGACACG ACTTATCGCC ACTGGCAGCA GCCACTGGTA ACAGGATTAG

4861 CAGAGCGAGG TATGTAGGCG GTGCTACAGA GTTCTTGAAG TGGTGGCCTA ACTACGGCTA

4921 CACTAGAAGA ACAGTATTTG GTATCTGCGC TCTGCTGAAG CCAGTTACCT TCGGAAAAAG

4981 AGTTGGTAGC TCTTGATCCG GCAAACAAAC CACCGCTGGT AGCGGTGGTT TTTTTGTTTG

5041 CAAGCAGCAG ATTACGCGCA GAAAAAAAGG ATCTCAAGAA GATCCTTTGA TCTTTTCTAC

5101 GGGGTCTGAC GCTCAGTGGA ACGAAAACTC ACGTTAAGGG ATTTTGGTCA TGAGATTATC

5161 AAAAAGGATC TTCACCTAGA TCCTTTTAAA TTAAAAATGA AGTTTTAAAT CAATCTAAAG

5221 TATATATGAG TAAACTTGGT CTGACAGTTA CCAATGCTTA ATCAGTGAGG CACCTATCTC

5281 AGCGATCTGT CTATTTCGTT CATCCATAGT TGCCTGACTC CCCGTCGTGT AGATAACTAC

5341 GATACGGGAG GGCTTACCAT CTGGCCCCAG TGCTGCAATG ATACCGCGAG ACCCACGCTC

5401 ACCGGCTCCA GATTTATCAG CAATAAACCA GCCAGCCGGA AGGGCCGAGC GCAGAAGTGG

5461 TCCTGCAACT TTATCCGCCT CCATCCAGTC TATTAATTGT TGCCGGGAAG CTAGAGTAAG

5521 TAGTTCGCCA GTTAATAGTT TGCGCAACGT TGTTGCCATT GCTACAGGCA TCGTGGTGTC

5581 ACGCTCGTCG TTTGGTATGG CTTCATTCAG CTCCGGTTCC CAACGATCAA GGCGAGTTAC

5641 ATGATCCCCC ATGTTGTGCA AAAAAGCGGT TAGCTCCTTC GGTCCTCCGA TCGTTGTCAG

5701 AAGTAAGTTG GCCGCAGTGT TATCACTCAT GGTTATGGCA GCACTGCATA ATTCTCTTAC

5761 TGTCATGCCA TCCGTAAGAT GCTTTTCTGT GACTGGTGAG TACTCAACCA AGTCATTCTG

5821 AGAATAGTGT ATGCGGCGAC CGAGTTGCTC TTGCCCGGCG TCAATACGGG ATAATACCGC

5881 GCCACATAGC AGAACTTTAA AAGTGCTCAT CATTGGAAAA CGTTCTTCGG GGCGAAAACT

5941 CTCAAGGATC TTACCGCTGT TGAGATCCAG TTCGATGTAA CCCACTCGTG CACCCAACTG

6001 ATCTTCAGCA TCTTTTACTT TCACCAGCGT TTCTGGGTGA GCAAAAACAG GAAGGCAAAA

6061 TGCCGCAAAA AAGGGAATAA GGGCGACACG GAAATGTTGA ATACTCATAC TCTTCCTTTT

6121 TCAATATTAT TGAAGCATTT ATCAGGGTTA TTGTCTCATG AGCGGATACA TATTTGAATG

6181 TATTTAGAAA AATAAACAAA TAGGGGTTCC GCGCACATTT CCCCGAAAAG TGCCACCTGA

6241 CGTC

//

LOCUS VEGFA165b_orf_3UTR_pcDNA3.1(+) 6178 bp DNA circular 31-JUL-2014

FEATURES Location/Qualifiers

promoter 863..881

/label="T7_promoter"

promoter complement(6084..6112)

/label="AmpR_promoter"

promoter 2620..2822

/label="SV40_promoter"

terminator 1772..1999

/label="bGH_PA_terminator"

CDS complement(5182..6042)

/label="Amp(R)"

CDS 2874..3674

/label="Neo(R)"

rep_origin complement(4408..5027)

/label="pBR322_origin"

rep_origin 2667..2744

/label="SV40_origin"

rep_origin 2062..2368

/label="f1_origin"

primer complement(4017..4035)

/label="M13_reverse_primer"

primer complement(1769..1786)

/label="BGH\rev\primer"

primer 769..789

/label="CMV_fwd_primer"

primer 3942..3961

/label="EBV_rev_primer"

primer complement(4034..4056)

/label="M13_pUC_rev_primer"

primer complement(2482..2502)

/label="pBABE_3_primer"

primer 2729..2748

/label="SV40pro_F_primer"

polyA_site 1775..1999

/label="BGH\pA"

promoter 308..818

/label="CMV_Promoter"

terminator 3856..3972

/label="SV40_PA_terminator"

promoter 236..852

/label="CMV_immearly_promoter"

CDS 2883..3677

/label="Neo_Kana"

CDS 911..1704

/label="VEGFA165b_orf_3UTR"

ORIGIN

1 GACGGATCGG GAGATCTCCC GATCCCCTAT GGTGCACTCT CAGTACAATC TGCTCTGATG

61 CCGCATAGTT AAGCCAGTAT CTGCTCCCTG CTTGTGTGTT GGAGGTCGCT GAGTAGTGCG

121 CGAGCAAAAT TTAAGCTACA ACAAGGCAAG GCTTGACCGA CAATTGCATG AAGAATCTGC

181 TTAGGGTTAG GCGTTTTGCG CTGCTTCGCG ATGTACGGGC CAGATATACG CGTTGACATT

241 GATTATTGAC TAGTTATTAA TAGTAATCAA TTACGGGGTC ATTAGTTCAT AGCCCATATA

301 TGGAGTTCCG CGTTACATAA CTTACGGTAA ATGGCCCGCC TGGCTGACCG CCCAACGACC

361 CCCGCCCATT GACGTCAATA ATGACGTATG TTCCCATAGT AACGCCAATA GGGACTTTCC

421 ATTGACGTCA ATGGGTGGAG TATTTACGGT AAACTGCCCA CTTGGCAGTA CATCAAGTGT

481 ATCATATGCC AAGTACGCCC CCTATTGACG TCAATGACGG TAAATGGCCC GCCTGGCATT

541 ATGCCCAGTA CATGACCTTA TGGGACTTTC CTACTTGGCA GTACATCTAC GTATTAGTCA

601 TCGCTATTAC CATGGTGATG CGGTTTTGGC AGTACATCAA TGGGCGTGGA TAGCGGTTTG

661 ACTCACGGGG ATTTCCAAGT CTCCACCCCA TTGACGTCAA TGGGAGTTTG TTTTGGCACC

721 AAAATCAACG GGACTTTCCA AAATGTCGTA ACAACTCCGC CCCATTGACG CAAATGGGCG

781 GTAGGCGTGT ACGGTGGGAG GTCTATATAA GCAGAGCTCT CTGGCTAACT AGAGAACCCA

841 CTGCTTACTG GCTTATCGAA ATTAATACGA CTCACTATAG GGAGACCCAA GCTGGCTAGC

901 GTTTAAACTT AAGCTTATGA ACTTTCTGCT GTCTTGGGTG CATTGGAGCC TTGCCTTGCT

961 GCTCTACCTC CACCATGCCA AGTGGTCCCA GGCTGCACCC ATGGCAGAAG GAGGAGGGCA

1021 GAATCATCAC GAAGTGGTGA AGTTCATGGA TGTCTATCAG CGCAGCTACT GCCATCCAAT

1081 CGAGACCCTG GTGGACATCT TCCAGGAGTA CCCTGATGAG ATCGAGTACA TCTTCAAGCC

1141 ATCCTGTGTG CCCCTGATGC GATGCGGGGG CTGCTGCAAT GACGAGGGCC TGGAGTGTGT

1201 GCCCACTGAG GAGTCCAACA TCACCATGCA GATTATGCGG ATCAAACCTC ACCAAGGCCA

1261 GCACATAGGA GAGATGAGCT TCCTACAGCA CAACAAATGT GAATGCAGAC CAAAGAAAGA

1321 TAGAGCAAGA CAAGAAAATC CCTGTGGGCC TTGCTCAGAG CGGAGAAAGC ATTTGTTTGT

1381 ACAAGATCCG CAGACGTGTA AATGTTCCTG CAAAAACACA GACTCGCGTT GCAAGGCGAG

1441 GCAGCTTGAG TTAAACGAAC GTACTTGCAG ATCTCTCACC AGGAAAGACT GATACAGAAC

1501 GATCGATACA GAAACCACGC TGCCGCCACC ACACCATCAC CATCGACAGA ACAGTCCTTA

1561 ATCCAGAAAC CTGAAATGAA GGAAGAGGAG ACTCTGCGCA GAGCACTTTG GGTCCGGAGG

1621 GCGAGACTCC GGCGGAAGCA TTCCCGGGCG GGTGACCCAG CACGGTCCCT CTTGGAATTG

1681 GATTCGCCAT TTTATTTTGA ATTCTGCAGA TATCCAGCAC AGTGGCGGCC GCTCGAGTCT

1741 AGAGGGCCCG TTTAAACCCG CTGATCAGCC TCGACTGTGC CTTCTAGTTG CCAGCCATCT

1801 GTTGTTTGCC CCTCCCCCGT GCCTTCCTTG ACCCTGGAAG GTGCCACTCC CACTGTCCTT

1861 TCCTAATAAA ATGAGGAAAT TGCATCGCAT TGTCTGAGTA GGTGTCATTC TATTCTGGGG

1921 GGTGGGGTGG GGCAGGACAG CAAGGGGGAG GATTGGGAAG ACAATAGCAG GCATGCTGGG

1981 GATGCGGTGG GCTCTATGGC TTCTGAGGCG GAAAGAACCA GCTGGGGCTC TAGGGGGTAT

2041 CCCCACGCGC CCTGTAGCGG CGCATTAAGC GCGGCGGGTG TGGTGGTTAC GCGCAGCGTG

2101 ACCGCTACAC TTGCCAGCGC CCTAGCGCCC GCTCCTTTCG CTTTCTTCCC TTCCTTTCTC

2161 GCCACGTTCG CCGGCTTTCC CCGTCAAGCT CTAAATCGGG GGCTCCCTTT AGGGTTCCGA

2221 TTTAGTGCTT TACGGCACCT CGACCCCAAA AAACTTGATT AGGGTGATGG TTCACGTAGT

2281 GGGCCATCGC CCTGATAGAC GGTTTTTCGC CCTTTGACGT TGGAGTCCAC GTTCTTTAAT

2341 AGTGGACTCT TGTTCCAAAC TGGAACAACA CTCAACCCTA TCTCGGTCTA TTCTTTTGAT

2401 TTATAAGGGA TTTTGCCGAT TTCGGCCTAT TGGTTAAAAA ATGAGCTGAT TTAACAAAAA

2461 TTTAACGCGA ATTAATTCTG TGGAATGTGT GTCAGTTAGG GTGTGGAAAG TCCCCAGGCT

2521 CCCCAGCAGG CAGAAGTATG CAAAGCATGC ATCTCAATTA GTCAGCAACC AGGTGTGGAA

2581 AGTCCCCAGG CTCCCCAGCA GGCAGAAGTA TGCAAAGCAT GCATCTCAAT TAGTCAGCAA

2641 CCATAGTCCC GCCCCTAACT CCGCCCATCC CGCCCCTAAC TCCGCCCAGT TCCGCCCATT

2701 CTCCGCCCCA TGGCTGACTA ATTTTTTTTA TTTATGCAGA GGCCGAGGCC GCCTCTGCCT

2761 CTGAGCTATT CCAGAAGTAG TGAGGAGGCT TTTTTGGAGG CCTAGGCTTT TGCAAAAAGC

2821 TCCCGGGAGC TTGTATATCC ATTTTCGGAT CTGATCAAGA GACAGGATGA GGATCGTTTC

2881 GCATGATTGA ACAAGATGGA TTGCACGCAG GTTCTCCGGC CGCTTGGGTG GAGAGGCTAT

2941 TCGGCTATGA CTGGGCACAA CAGACAATCG GCTGCTCTGA TGCCGCCGTG TTCCGGCTGT

3001 CAGCGCAGGG GCGCCCGGTT CTTTTTGTCA AGACCGACCT GTCCGGTGCC CTGAATGAAC

3061 TGCAGGACGA GGCAGCGCGG CTATCGTGGC TGGCCACGAC GGGCGTTCCT TGCGCAGCTG

3121 TGCTCGACGT TGTCACTGAA GCGGGAAGGG ACTGGCTGCT ATTGGGCGAA GTGCCGGGGC

3181 AGGATCTCCT GTCATCTCAC CTTGCTCCTG CCGAGAAAGT ATCCATCATG GCTGATGCAA

3241 TGCGGCGGCT GCATACGCTT GATCCGGCTA CCTGCCCATT CGACCACCAA GCGAAACATC

3301 GCATCGAGCG AGCACGTACT CGGATGGAAG CCGGTCTTGT CGATCAGGAT GATCTGGACG

3361 AAGAGCATCA GGGGCTCGCG CCAGCCGAAC TGTTCGCCAG GCTCAAGGCG CGCATGCCCG

3421 ACGGCGAGGA TCTCGTCGTG ACCCATGGCG ATGCCTGCTT GCCGAATATC ATGGTGGAAA

3481 ATGGCCGCTT TTCTGGATTC ATCGACTGTG GCCGGCTGGG TGTGGCGGAC CGCTATCAGG

3541 ACATAGCGTT GGCTACCCGT GATATTGCTG AAGAGCTTGG CGGCGAATGG GCTGACCGCT

3601 TCCTCGTGCT TTACGGTATC GCCGCTCCCG ATTCGCAGCG CATCGCCTTC TATCGCCTTC

3661 TTGACGAGTT CTTCTGAGCG GGACTCTGGG GTTCGAAATG ACCGACCAAG CGACGCCCAA

3721 CCTGCCATCA CGAGATTTCG ATTCCACCGC CGCCTTCTAT GAAAGGTTGG GCTTCGGAAT

3781 CGTTTTCCGG GACGCCGGCT GGATGATCCT CCAGCGCGGG GATCTCATGC TGGAGTTCTT

3841 CGCCCACCCC AACTTGTTTA TTGCAGCTTA TAATGGTTAC AAATAAAGCA ATAGCATCAC

3901 AAATTTCACA AATAAAGCAT TTTTTTCACT GCATTCTAGT TGTGGTTTGT CCAAACTCAT

3961 CAATGTATCT TATCATGTCT GTATACCGTC GACCTCTAGC TAGAGCTTGG CGTAATCATG

4021 GTCATAGCTG TTTCCTGTGT GAAATTGTTA TCCGCTCACA ATTCCACACA ACATACGAGC

4081 CGGAAGCATA AAGTGTAAAG CCTGGGGTGC CTAATGAGTG AGCTAACTCA CATTAATTGC

4141 GTTGCGCTCA CTGCCCGCTT TCCAGTCGGG AAACCTGTCG TGCCAGCTGC ATTAATGAAT

4201 CGGCCAACGC GCGGGGAGAG GCGGTTTGCG TATTGGGCGC TCTTCCGCTT CCTCGCTCAC

4261 TGACTCGCTG CGCTCGGTCG TTCGGCTGCG GCGAGCGGTA TCAGCTCACT CAAAGGCGGT

4321 AATACGGTTA TCCACAGAAT CAGGGGATAA CGCAGGAAAG AACATGTGAG CAAAAGGCCA

4381 GCAAAAGGCC AGGAACCGTA AAAAGGCCGC GTTGCTGGCG TTTTTCCATA GGCTCCGCCC

4441 CCCTGACGAG CATCACAAAA ATCGACGCTC AAGTCAGAGG TGGCGAAACC CGACAGGACT

4501 ATAAAGATAC CAGGCGTTTC CCCCTGGAAG CTCCCTCGTG CGCTCTCCTG TTCCGACCCT

4561 GCCGCTTACC GGATACCTGT CCGCCTTTCT CCCTTCGGGA AGCGTGGCGC TTTCTCATAG

4621 CTCACGCTGT AGGTATCTCA GTTCGGTGTA GGTCGTTCGC TCCAAGCTGG GCTGTGTGCA

4681 CGAACCCCCC GTTCAGCCCG ACCGCTGCGC CTTATCCGGT AACTATCGTC TTGAGTCCAA

4741 CCCGGTAAGA CACGACTTAT CGCCACTGGC AGCAGCCACT GGTAACAGGA TTAGCAGAGC

4801 GAGGTATGTA GGCGGTGCTA CAGAGTTCTT GAAGTGGTGG CCTAACTACG GCTACACTAG

4861 AAGAACAGTA TTTGGTATCT GCGCTCTGCT GAAGCCAGTT ACCTTCGGAA AAAGAGTTGG

4921 TAGCTCTTGA TCCGGCAAAC AAACCACCGC TGGTAGCGGT GGTTTTTTTG TTTGCAAGCA

4981 GCAGATTACG CGCAGAAAAA AAGGATCTCA AGAAGATCCT TTGATCTTTT CTACGGGGTC

5041 TGACGCTCAG TGGAACGAAA ACTCACGTTA AGGGATTTTG GTCATGAGAT TATCAAAAAG

5101 GATCTTCACC TAGATCCTTT TAAATTAAAA ATGAAGTTTT AAATCAATCT AAAGTATATA

5161 TGAGTAAACT TGGTCTGACA GTTACCAATG CTTAATCAGT GAGGCACCTA TCTCAGCGAT

5221 CTGTCTATTT CGTTCATCCA TAGTTGCCTG ACTCCCCGTC GTGTAGATAA CTACGATACG

5281 GGAGGGCTTA CCATCTGGCC CCAGTGCTGC AATGATACCG CGAGACCCAC GCTCACCGGC

5341 TCCAGATTTA TCAGCAATAA ACCAGCCAGC CGGAAGGGCC GAGCGCAGAA GTGGTCCTGC

5401 AACTTTATCC GCCTCCATCC AGTCTATTAA TTGTTGCCGG GAAGCTAGAG TAAGTAGTTC

5461 GCCAGTTAAT AGTTTGCGCA ACGTTGTTGC CATTGCTACA GGCATCGTGG TGTCACGCTC

5521 GTCGTTTGGT ATGGCTTCAT TCAGCTCCGG TTCCCAACGA TCAAGGCGAG TTACATGATC

5581 CCCCATGTTG TGCAAAAAAG CGGTTAGCTC CTTCGGTCCT CCGATCGTTG TCAGAAGTAA

5641 GTTGGCCGCA GTGTTATCAC TCATGGTTAT GGCAGCACTG CATAATTCTC TTACTGTCAT

5701 GCCATCCGTA AGATGCTTTT CTGTGACTGG TGAGTACTCA ACCAAGTCAT TCTGAGAATA

5761 GTGTATGCGG CGACCGAGTT GCTCTTGCCC GGCGTCAATA CGGGATAATA CCGCGCCACA

5821 TAGCAGAACT TTAAAAGTGC TCATCATTGG AAAACGTTCT TCGGGGCGAA AACTCTCAAG

5881 GATCTTACCG CTGTTGAGAT CCAGTTCGAT GTAACCCACT CGTGCACCCA ACTGATCTTC

5941 AGCATCTTTT ACTTTCACCA GCGTTTCTGG GTGAGCAAAA ACAGGAAGGC AAAATGCCGC

6001 AAAAAAGGGA ATAAGGGCGA CACGGAAATG TTGAATACTC ATACTCTTCC TTTTTCAATA

6061 TTATTGAAGC ATTTATCAGG GTTATTGTCT CATGAGCGGA TACATATTTG AATGTATTTA

6121 GAAAAATAAA CAAATAGGGG TTCCGCGCAC ATTTCCCCGA AAAGTGCCAC CTGACGTC

//
